# High-Resolution Longitudinal Dynamics of the Cystic Fibrosis Sputum Microbiome and Metabolome through Antibiotic Therapy

**DOI:** 10.1128/mSystems.00292-20

**Published:** 2020-06-23

**Authors:** Ruma Raghuvanshi, Karla Vasco, Yoshiki Vázquez-Baeza, Lingjing Jiang, James T. Morton, Danxun Li, Antonio Gonzalez, Lindsay DeRight Goldasich, Gregory Humphrey, Gail Ackermann, Austin D. Swafford, Douglas Conrad, Rob Knight, Pieter C. Dorrestein, Robert A. Quinn

**Affiliations:** a Department of Biochemistry and Molecular Biology, Michigan State University, East Lansing, Michigan, USA; b Department of Pediatrics, University of California San Diego, La Jolla, California, USA; c Center for Microbiome Innovation, University of California San Diego, La Jolla, California, USA; d Jacobs School of Engineering, University of California San Diego, La Jolla, California, USA; e Division of Biostatistics, University of California San Diego, La Jolla, California, USA; f Center for Computational Biology, Flatiron Institute, Simons Foundation, New York, New York, USA; g Department of Medicine, University of California San Diego, La Jolla, California, USA; h Department of Computer Science and Engineering and Bioengineering, University of California San Diego, La Jolla, California, USA; i Skaggs School of Pharmacy and Pharmaceutical Sciences, University of California San Diego, La Jolla, California, USA; Mayo Clinic

**Keywords:** cystic fibrosis, microbiome, antibiotics, metabolome

## Abstract

Microbial diversity in the cystic fibrosis (CF) lung decreases over decades as pathogenic bacteria such as Pseudomonas aeruginosa take over. The dynamics of the CF microbiome and metabolome over shorter time frames, however, remain poorly studied. Here, we analyze paired microbiome and metabolome data from 594 sputum samples collected over 401 days from six adult CF subjects (subject mean = 179 days) through periods of clinical stability and 11 CF pulmonary exacerbations (CFPE). While microbiome profiles were personalized (permutational multivariate analysis of variance [PERMANOVA] *r*^2^ = 0.79, *P* < 0.001), we observed significant intraindividual temporal variation that was highest during clinical stability (linear mixed-effects [LME] model, *P* = 0.002). This included periods where the microbiomes of different subjects became highly similar (UniFrac distance, <0.05). There was a linear increase in the microbiome alpha-diversity and in the log ratio of anaerobes to pathogens with time (*n* = 14 days) during the development of a CFPE (LME *P* = 0.0045 and *P* = 0.029, respectively). Collectively, comparing samples across disease states showed there was a reduction of these two measures during antibiotic treatment (LME *P* = 0.0096 and *P* = 0.014, respectively), but the stability data and CFPE data were not significantly different from each other. Metabolome alpha-diversity was higher during CFPE than during stability (LME *P* = 0.0085), but no consistent metabolite signatures of CFPE across subjects were identified. Virulence-associated metabolites from P. aeruginosa were temporally dynamic but were not associated with any disease state. One subject died during the collection period, enabling a detailed look at changes in the 194 days prior to death. This subject had over 90% *Pseudomonas* in the microbiome at the beginning of sampling, and that level gradually increased to over 99% prior to death. This study revealed that the CF microbiome and metabolome of some subjects are dynamic through time. Future work is needed to understand what drives these temporal dynamics and if reduction of anaerobes correlate to clinical response to CFPE therapy.

**IMPORTANCE** Subjects with cystic fibrosis battle polymicrobial lung infections throughout their lifetime. Although antibiotic therapy is a principal treatment for CF lung disease, we have little understanding of how antibiotics affect the CF lung microbiome and metabolome and how much the community changes on daily timescales. By analyzing 594 longitudinal CF sputum samples from six adult subjects, we show that the sputum microbiome and metabolome are dynamic. Significant changes occur during times of stability and also through pulmonary exacerbations (CFPEs). Microbiome alpha-diversity increased as a CFPE developed and then decreased during treatment in a manner corresponding to the reduction in the log ratio of anaerobic bacteria to classic pathogens. Levels of metabolites from the pathogen P. aeruginosa were also highly variable through time and were negatively associated with anaerobes. The microbial dynamics observed in this study may have a significant impact on the outcome of antibiotic therapy for CFPEs and overall subject health.

## Abstract (precis)

Subjects with cystic fibrosis battle polymicrobial lung infections throughout their lifetime. Although antibiotic therapy is a principal treatment for CF lung disease, we have little understanding of how antibiotics affect the CF lung microbiome and metabolome and how much the community changes on daily timescales. By analyzing 594 longitudinal CF sputum samples from six adult subjects, we show that the sputum microbiome and metabolome are dynamic. Significant changes occur during times of stability and also through pulmonary exacerbations (CFPEs). Microbiome alpha-diversity increased as a CFPE developed and then decreased during treatment in a manner corresponding to the reduction in the log ratio of anaerobic bacteria to classic pathogens. Levels of metabolites from the pathogen P. aeruginosa were also highly variable through time and were negatively associated with anaerobes. The microbial dynamics observed in this study may have a significant impact on the outcome of antibiotic therapy for CFPEs and overall subject health.

## INTRODUCTION

The respiratory tracts of individuals with cystic fibrosis (CF) are colonized by a polymicrobial community that impacts the pathology and progression of the disease (1–3). The microbiome of sputum expectorated from the lung includes opportunistic pathogens, such as Pseudomonas aeruginosa, Staphylococcus aureus, Stenotrophomonas maltophilia, Burkholderia cepacia, and Achromobacter xylosoxidans, but a myriad of lesser understood oral anaerobes are also detected (1–3). It is known that pathogens come to dominate the community profiles as subjects age and microbial diversity decreases (1), yet we have a poor understanding of these dynamics in shorter longitudinal time frames, such as throughout CF pulmonary exacerbations (CFPEs). Some studies have reported changes occurring during CFPE (4–9), primarily via reduction in the relative abundance of rare taxa during treatment, particularly anaerobic bacteria (*Prevotella*, *Veillonella*, *Gemella*, etc.) (4, 5, 9–11), but whether this represents a dysbiotic shift in the CF microbiome or regular changes in microbial dynamics without clinical relevance remains unknown. In other studies, the CF microbiome was found to remain relatively static through CFPE (8, 12–14), complicating our understanding of microbiome dynamics. The CF microbiome has also been shown to be highly personalized (1, 11, 15), but it is unknown whether this personalization is maintained over shorter longitudinal time frames or if the communities are dynamic.

Recent studies have begun to examine the metabolome of CF sputum, comprised of DNA, mucins, surfactant, and a myriad of small molecules from microbial, host, and xenobiotic sources that are highly personalized (16) and have important implications for disease pathology (17–20). Virulence-associated metabolites from P. aeruginosa are also detected, as well as fermentation metabolites from streptococci (21). A recent study showed that as lung function declines, subjects accumulate more peptides and amino acids in their sputum (17), which are derived from neutrophil elastase activity in response to microbial infections. The contents of the CF metabolome are highly diverse, but how this chemistry changes through time and around CFPE events is virtually unknown.

This report presents a high-resolution analysis of the longitudinal dynamics of the adult CF sputum metabolome and microbiome. Sputum samples were collected from six subjects in their homes as frequently as possible for a period of 401 days. Eleven CFPE events were captured through the sampling period, providing detailed insight into the microbial and chemical changes in sputum through these important clinical events.

## RESULTS

### Longitudinal sampling and microbiome and metabolome data generation from CF sputum samples.

A total of 594 sputum samples were self-collected by six CF subjects (CF066, CF146, CF176, CF189, CF318, and CF353) with declining lung function and health over a total of 401 days (subject mean = 179 sampled days, range = 22 to 190) (see Fig. S1 in the supplemental material; see also Data Set S1, sheets 1 and 2, in the supplemental material). Subjects did not all begin sampling on the same day but were asked to collect as frequently as possible during their collection period, resulting in varied sample numbers per subject (Fig. S1; see also Data Set S1, sheets 1 and 2). All experienced at least one period of CFPE and treatment during the study (Data Set S1, sheets 1 and 2). Samples were classified as “CFPE” samples if they were collected within 14 days prior to treatment, as “treatment” samples if they were collected during the 21 days of treatment, and as “stable” if they were collected outside those periods. All samples were delivered to the clinic by each subject after storage in a home freezer, with the exception of the last 22 samples from one subject (CF176) who died during the study. Paired 16S rRNA gene sequencing and untargeted metabolomic data were generated to evaluate the microbial community and chemical composition of these sputum samples.

10.1128/mSystems.00292-20.1 FIG S1 Longitudinal collection of sputum samples from this study and antibiotic treatment events from each of the six subjects. (a) Sample collection with antibiotic treatments highlighted. The time in days is shown on a linear scale for each graph; dashes represent sputum samples collected, and gaps in the collection are shown as empty spaces. (b) Overview of sample collection with different disease states. Points in the longitudinal graph represent individual samples, and the line denotes disease state through time. Download FIG S1, PDF file, 0.3 MB. Copyright © 2020 Raghuvanshi et al. 2020 Raghuvanshi et al. This content is distributed under the terms of the Creative Commons Attribution 4.0 International license.

10.1128/mSystems.00292-20.9 DATA SET S1 (Sheet 1) (a) List of subjects and the clinical information associated with this study. (b) Summary of samples collected by subject and the disease state represented by each sample. (Sheet 2) List of samples and associated clinical data from subjects in this study. (Sheet 3) Classification of ASVs in the microbiome data as pathogens or anaerobes. (Sheet 4) Quantitation of the percentages of microbiome weighted UniFrac distances between subjects CF146 and CF186 compared to the CF176 reference that were below 0.05. (Sheet 5) Difference between exacerbation and treatment periods with respect to Shannon diversity from the linear-spline mixed-effects model. (Sheet 6) Confusion matrix of random forest model run on the metabolomic data according to disease state classification. The overall out-of-bag error rate of the model was 27.5%. (Sheet 7) Number of 16S rRNA gene reads for all sputum and blank control samples in this study. (Sheet 8) Annotations of spectral matches to GNPS libraries for metabolites of interest in the sputum data. (Sheet 9) Conditional probabilities of metabolite and microbial associations from MMvec. Download Data Set S1, XLSX file, 0.9 MB. Copyright © 2020 Raghuvanshi et al. 2020 Raghuvanshi et al. This content is distributed under the terms of the Creative Commons Attribution 4.0 International license.

As expected, the microbiome contained a mixture of classic CF pathogens and oral anaerobic bacteria (classified according to Data Set S1, sheet 3). The metabolomic data included molecules from host cells, microbial cells, and xenobiotics (Fig. S2). There were 4,988 unique spectra detected in the sputum samples, with 394 annotations from the Global Natural Products Social Molecular Networking (GNPS) mass spectral libraries (7.9%) (22). The most prevalent known molecules were phospholipids, sphingolipids, and antibiotics (Fig. S2).

10.1128/mSystems.00292-20.2 FIG S2 (a) Metabolite identifications and proportions in each subject as a percentage of the total molecular features (left panel) and the only known compounds (right panel). (b) Distribution of the number of samples and reads per sample from the 16S rRNA gene amplicon sequencing. Small blue dashes along the *x* axis represent individual sample, and the corresponding number of reads; the larger blue boxes represent histograms of the number of reads and number of samples. Download FIG S2, PDF file, 0.4 MB. Copyright © 2020 Raghuvanshi et al. 2020 Raghuvanshi et al. This content is distributed under the terms of the Creative Commons Attribution 4.0 International license.

### Sputum microbiome and metabolome are largely subject specific.

**FIG 1:**
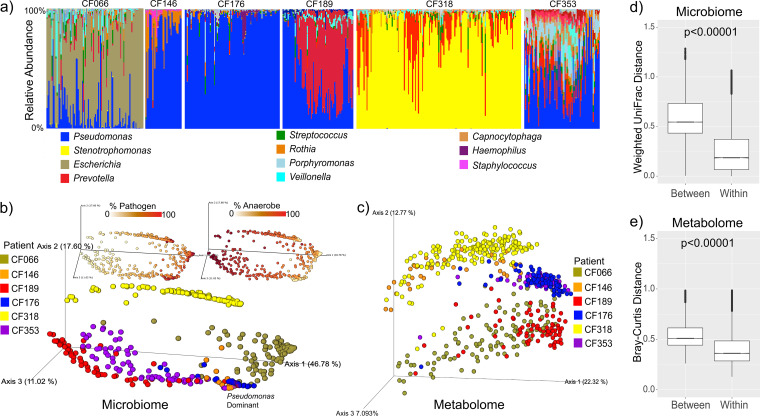
(a) Bar plots representing the microbiome of sputum samples from the six subjects plotted chronologically through the collection. Gaps in sample collection are not shown. (b) PCoA plot of the weighted UniFrac distances of the microbiome data colored by subject. Inset are the samples colored on a scale representing the percentage of the anaerobe or percentage of the pathogen as defined in Data Set S1, sheet 3. Samples where *Pseudomonas* is the dominant ASV in the plot are highlighted. (c) PCoA plot of the Bray-Curtis distance of the metabolomic data, including all detected metabolite features colored by subject. (d and e) Within-subject and between-subject distances of microbiome (weighted UniFrac distance) data (d) and metabolome (Bray-Curtis distance) data (e). Significance was tested with an LME model with subject as a fixed effect.

Four of the six subjects had *Pseudomonas* as the most abundant classic pathogen in their sputa, while the other two were infected with *Stenotrophomonas* (CF318) or *Escherichia* (CF066) (Fig. 1a). Principal-coordinate analysis (PCoA) of the beta-diversity between samples showed that both the sputum microbiome and the sputum metabolome were highly individualized (weighted UniFrac distances for microbes, permutational multivariate analysis of variance [PERMANOVA] by subject F = 30.48, *r*^2^ = 0.79, *P* = 0.001; Bray-Curtis distances for metabolites, PERMANOVA by subject F = 24.81, *r*^2^ = 0.372, *P* = 0.001) (Fig. 1b and c). There was greater beta-diversity variation across subjects than within subjects for both data types (Fig. 1d and e). Alpha-diversities of the microbiome and metabolome were also individualized and were significantly different based on subject source (except CF146 and CF318; Fig. S3a and b; see also Fig. S4). CF176 had the lowest microbiome alpha-diversity (Fig. S3a) but the highest metabolome alpha-diversity (Fig. S3b), a contrasting phenomenon similar to that reported from a previous CF sputum multi-omics study (17). Despite the overall personalization, four of the subjects developed highly similar microbiomes at times during the dense longitudinal sampling period (CF176, CF146, CF353, and CF189, Fig. 1b and c). Comparing to CF176 as a reference, 23.0% of samples from CF146, 9.7% from CF189, and 1.3% from CF353 had a weighted UniFrac distance value of less than 0.05 (Fig. S5a; see also Data Set S1, sheet 4), indicating almost identical microbial communities. The metabolome data had no samples where the Bray-Curtis distance value was below 0.05, indicating stronger personalization in this data set as a whole; however, CF176 and CF353 showed similarity in the PCoA plot with a mean Bray-Curtis distance value of 0.39 (Fig. 1b and c; see also Fig. S5b).

10.1128/mSystems.00292-20.3 FIG S3 (a) Shannon diversity distributions of the sputum microbiome samples within each subject. (b) Shannon diversity distributions of the metabolome. Data were calculated for all molecular features identified using the mzMine software. (c) Weighted UniFrac distances of all pairwise microbiome samples compared to each other within the same subject. Also shown are data representing the weighted UniFrac distances from a recently published cross-sectional cohort (*n* = 88) (17). (d) Bray-Curtis distances of all pairwise metabolome samples compared to each other within the same subject. Also shown are data representing the Bray-Curtis distances from a recently published cross-sectional cohort (*n* = 88) (17). The statistical tests represented are the Kruskall-Wallis test with overall *P* value corresponding to the comparisons across subjects. *P* values corresponding to all pairwise comparisons are shown below the threshold above each figure unless indicated otherwise. XSect = cross-sectional study. Download FIG S3, PDF file, 0.7 MB. Copyright © 2020 Raghuvanshi et al. 2020 Raghuvanshi et al. This content is distributed under the terms of the Creative Commons Attribution 4.0 International license.

10.1128/mSystems.00292-20.4 FIG S4 (a) Line plots of the Shannon index and (b) log ratios of anaerobes to pathogens in each subject through time, with box plots classified by disease state. The date immediately prior to antibiotic therapy is highlighted with an asterisk. Download FIG S4, PDF file, 0.6 MB. Copyright © 2020 Raghuvanshi et al. 2020 Raghuvanshi et al. This content is distributed under the terms of the Creative Commons Attribution 4.0 International license.

10.1128/mSystems.00292-20.5 FIG S5 Microbiome and metabolome beta-diversity comparisons to CF176. Bray-Curtis dissimilarities are calculated for the metabolome data and weighted UniFrac distances for the microbiome data for each subject compared to all samples from CF176 as a reference. Download FIG S5, PDF file, 9.1 MB. Copyright © 2020 Raghuvanshi et al. 2020 Raghuvanshi et al. This content is distributed under the terms of the Creative Commons Attribution 4.0 International license.

### Microbiome and metabolome of sputum are dynamic in short time frames.

To better characterize intraindividual dynamics, we quantified multi-omic variation as the percentage of samples within each subject with values that were greater than 1.5× their beta-diversity interquartile range (IQR) (analogous to the analysis by Caverly et al. [23]) and the incidence of samples that had a weighted UniFrac or Bray-Curtis distance value above 0.6 (a cutoff to represent a microbiome that was highly differentiated with respect to 16S or metabolomic data, respectively). To provide a reference frame for comparison, we computed the same metrics for variation in samples from a recently published cross-sectional study (*n* = 88) (17; Fig. S3c and d; see also Video S1 in the supplemental material). In our cohort, 23.8% of the microbiomes within an individual were outside their IQR (Table 1), a value similar to the 24.4% of samples across individuals seen in the cross-sectional study (Fig. S3c). The within-subject weighted UniFrac distance values were above 0.6 for 10.5% of the longitudinal comparisons, compared to 45.6% across individuals in the cross-sectional study (Fig. S3c). The incidence of these samples with high beta-diversity were most common in two subjects with *Pseudomonas* as the pathogen with the highest relative abundance (CF189 [30.5%] and CF353 [12.1%]), while the end-stage subject (CF176) had no samples above this distance threshold and the lowest microbial variation through time (Fig. 1b and c; see also Fig. S3c) (Table 1). Similarly, the mean proportion of metabolome sample comparisons with values 1.5× outside their IQR was 26.6% (28.0% in the cross-sectional study), with 12.7% having a Bray-Curtis distance above 0.6 (56.4% in the cross-sectional study) (Table 1; see also Fig. S3d). Two of the *Pseudomonas*-dominated subjects, CF176 and CF353, showed little change in their sputum metabolome, with <3% of samples having values above the Bray-Curtis distance value of 0.6 (Fig. S3d). Collectively, the alpha- and beta-diversity results demonstrate that although there was strong personalization in the overall microbiome profiles, the communities within five of the six individuals were dynamic and driven by changes in the relative abundances of anaerobes and dominant pathogens, such as *Pseudomonas* or *Stenotrophomonas* (Fig. 1a; see also the animated video [24] showing changes through time [see Video S1 in the supplemental material]).

**TABLE 1:** Microbiome (UniFrac distance) and metabolome (Bray-Curtis distance) variation in the different subjects through time^*a*^

Category and subject ID	% outside IQR	% above 0.6	No. of comparisons
Microbiome			
CF66	24.512	8.902	4,561
CF146	33.068	2.703	629
CF176	10.297	0.023	4,370
CF189	25.062	30.53	2,414
CF318	22.96	8.742	13,040
CF353	27.185	12.185	2,700
Avg	23.848	10.514	
Cross-sectional	24.4	45.6	10,278

Metabolome			
CF66	25.961	32.822	5,778
CF146	20.509	20.509	629
CF176	33.37	2.632	4,558
CF189	30.655	7.995	2,414
CF318	21.707	9.87	13,899
CF353	27.509	2.501	3,159
Avg	26.618	12.721	
Cross-sectional	28	56.4	10,278

a All samples were compared to all others, and the percentages of comparisons outside the interquartile range (IQR), as well as the number of comparisons with a beta-diversity distance value above 0.6, are reported.

### Multi-omic variation around exacerbation.

**FIG 2:**
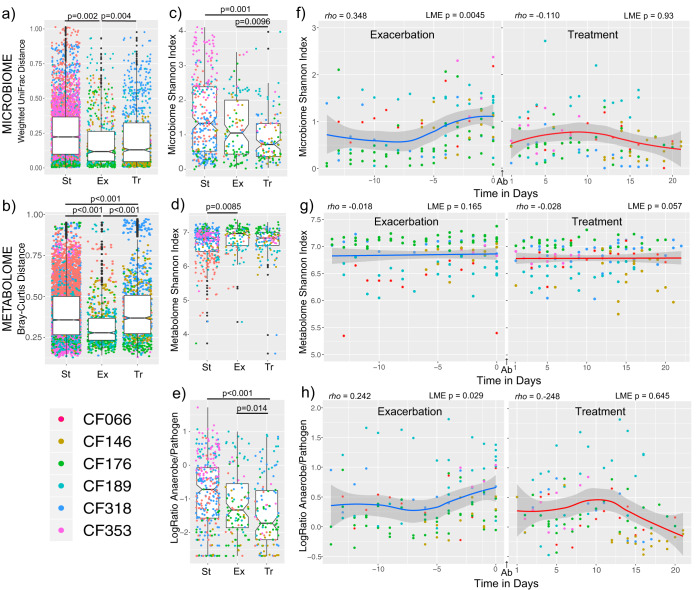
The microbiome and metabolome variation around CFPEs. (a and b) Notch plots of the (a) microbiome weighted UniFrac distances and (b) metabolome Bray-Curtis distances between samples classified as CFPE (−14 days from antibiotic treatment), treatment (during 21 days of antibiotic treatment), or stable (outside these time periods). Statistical significance across the class comparisons was tested using an LME model with subject as random effects and Tukey’s *post hoc* tests. (c and d) Shannon index of microbiome diversity (c) and metabolome diversity (d) in samples collected during different disease states. Statistical significance across the class comparisons was tested using an LME model with subject as random effects and Tukey’s *post hoc* tests. (e) Notch plots of the log ratios of anaerobes to pathogens in samples classified as CFPE, treatment, or stable. (Statistics are presented as described above). (f and g) Shannon index of microbiome diversity (f) and metabolome diversity (g) through the 14 days prior to a CFPE and the 21 days of treatment. Spearman’s *rho* and the corresponding *P* value from an LME model are shown for the regression with time in days. (h) Log ratio of anaerobes to pathogens through the 14 days prior to a CFPE and the 21 days of treatment. (Statistics are presented as described for panel f). Antibiotics were administered between day 0 and day 1 (denoted as “Ab” in panels f to h). All exacerbations for all subjects are shown in the plots.

To examine whether disease state (CFPE, treatment, or stable) was a primary driver of the dynamism seen, the population-level alpha-diversity and beta-diversity data from the microbial communities and metabolomes across disease states were compared. With subject source accounted for as a covariate, the beta-diversity values were significantly different based on disease state for the metabolomic data, though the level of variance explained by this parameter was low (PERMANOVA *r*^2^ = 0.032, *P* = 0.001). There was not a significant difference in beta-diversity based on disease state for the microbiome data (PERMANOVA *r*^2^ = 0.072, *P* = 0.223). Pairwise comparisons of the weighted UniFrac distance values within disease states from each subject using a linear mixed-effects (LME) model showed that the stable disease state had the highest degree of microbial variability (Fig. 2a). Metabolome variability was highest during the treatment period, followed by the stable period, and was lowest during CFPE (Fig. 2b, LME *P* < 0.001, all pairwise comparisons). Collectively, the alpha-diversity of the microbiome was significantly higher during the stability period than during the treatment period (LME *P* = 0.001) and during the CFPE period than during the treatment period (LME *P* = 0.0096), but the levels did not differ significantly between the stable and CFPE states (Fig. 2c). Alpha-diversity of the metabolome was highest during exacerbations, but this was only significant compared to the stable state (Fig. 2d, LME *P* = 0.0085). With the identification of lower microbial alpha-diversity during the treatment period, we further investigated these changes by comparing the log ratio of anaerobes to pathogens and found that this ratio was also significantly higher during stability and CFPE than during treatment but that the ratios did not differ significantly between the stable and CFPE states (Fig. 2e).

Because we had high-resolution longitudinal samples, we took a closer look at the microbial dynamics that had occurred through time during the development and treatment of the 11 CFPE events. The alpha-diversity of the microbial community significantly increased in the 14 days leading up to antibiotic therapy for a CFPE (Spearman’s *rho *= 0.348 with time in days, LME *P* = 0.0045, Fig. 2f), but there was no change in alpha diversity through time during the 21 days of treatment (Spearman’s *rho* = −0.110, LME *P* = 0.93, Fig. 2f). There were no significant changes in diversity measures of the metabolome through time during the CFPE period or the treatment period (Fig. 2g). The log ratio of anaerobes to pathogens also increased with time approaching the start of antibiotic treatment for a CFPE (*rho *= 0.242, LME *P* = 0.029) but did not change with time during the treatment period (*rho *= −0.248, LME *P* = 0.645, Fig. 2h). To further test the changes in microbial diversity that occurred with time through the CFPE and treatment periods, we also used an LME model to describe the temporal trajectories with a linear spline (or broken stick) and found that the Shannon diversity slope with time was significantly higher through CFPE than the treatment period (LME *P* = 0.008, Data Set S1, sheet 5).

To identify metabolites changing in the three disease states, we trained a random forest classification model (25) on data from the disease state to determine whether individual metabolites were altered. The random forest model did not indicate strong overall changes in the metabolome around exacerbation (out-of-bag error rate = 27.5%, Data Set S1, sheet 6). Variables of importance to the classification were primarily represented by antibiotics given to the subjects. Nonantibiotic metabolites of importance to the classification included stearoyl-l-carnitine and hemin; however, the corresponding data were not significantly different between the exacerbation and treatment states across subjects using an LME model (false-discovery-rate [FDR] corrected *q* > 0.05).

In light of the findings previously reported by Caverly et al. (23), who showed changes in bacterial diversity during times of stability due to maintenance therapies, and of the fact that we detected some maintenance antibiotics in the metabolomic data during stable periods, we analyzed the correlations between the abundances of azithromycin and trimethoprim (provided as maintenance therapies) and microbiome alpha-diversity. We found a negative correlation between microbiome Shannon diversity and the abundance of trimethoprim in the same samples during stability (linear mixed model [LMM] *P* = 0.0048, Spearman’s *rho* = −0.39) but not between microbiome Shannon diversity and the abundance of azithromycin (LMM *P* = 0.489, Spearman’s *rho* = 0.35, Fig. S6).

10.1128/mSystems.00292-20.6 FIG S6 Linear regression of the area-under-curve abundance of the maintenance therapy antibiotics azithromycin and trimethoprim during stable periods with Shannon index values determined for the 6 subjects. Each subject is plotted separately and highlighted by color. The *P* value result corresponding to an LMM result for the correlation with the subject as random effects and the Spearman’s *rho* are shown. Download FIG S6, PDF file, 0.3 MB. Copyright © 2020 Raghuvanshi et al. 2020 Raghuvanshi et al. This content is distributed under the terms of the Creative Commons Attribution 4.0 International license.

### Metabolite and microbiome associations.

We used a method of identifying metabolites associated with microbial amplicon sequence variants (ASVs) called microbial-metabolite vectors (MMvec; 26) to investigate the relationship of these molecules with a changing microbial community (Data Set S1, sheet 9). We gave particular attention to P. aeruginosa virulence-associated metabolites, including phenazines, rhamnolipids, and quinolones, that can have strong effects on other microbes in the community and host cells *in vitro* (24, 27, 28). We detected 36 different quinolones, eight rhamnolipids, and one siderophore (pyochelin) known to be produced by Pseudomonas aeruginosa in this longitudinal data set but detected no phenazines. At least one of these metabolites was found in four of the six subjects (CF066, CF176, CF353, and CF189, Fig. S7a), and all had *Pseudomonas* in their microbiome. Interestingly, none of these metabolites were detected in subject CF146, even though this subject’s microbiome had on average a 77.0% relative abundance of *Pseudomonas* through the collection, and CF353 had low to undetectable amounts of quinolones and rhamnolipids but an average of 41.0% *Pseudomonas*. The abundance of these metabolites was not associated with any disease state (LME *P* > 0.05). Using the MMvec method, we found that the quinolone 4-hydroxy-2-nonylquinolone (NHQ) had a high conditional log-probability of association with *Pseudomonas* in the data (1.18 *logP*, 98th percentile) whereas its related metabolite 4-hydroxy-2-heptylquinolone (HHQ) also showed a high level of association (1.035 *logP*, 96th percentile). These conditional probabilities did not correspond to strong linear associations of the relative abundance of *Pseudomonas* with the metabolite abundances (Fig. S7a), highlighting the importance of compositionally coherent approaches such as MMvec (29). However, the metabolites themselves, particularly the two quinolones and the rhamnolipids, were highly correlated with each other (Fig. S7a). Learning associations of metabolites with the other ASVs of interest showed a negative association between the *Pseudomonas* quinolones and anaerobes. NHQ and HHQ were highly negatively associated with *Streptococcus* (−4.34 *logP* and 99th percentile and −3.80 and 99th percentile, respectively), Veillonella parvula (−4.65 *logP* and 99th percentile and −4.06 and 99th percentile), and Prevotella melaninogenica (−3.51 *logP* and 99th percentile and −3.06 and 99th percentile) (Fig. S6b; see also Data Set S1, sheet 9).

10.1128/mSystems.00292-20.7 FIG S7 (a) Pairwise regressions between P. aeruginosa virulence-associated metabolites HHQ, NHQ, and the rhamnolipid Rha-C8-C8 and the relative abundance of *Pseudomonas* in the microbiome data. Pearson’s *r* for the correlation is shown in each plot. (b) Biplots of metabolite and microbial associations. Each ball in the EMPeror plot represents a metabolite, and the vectors represent the microbial ASVs associated with those metabolites. ASVs of interest with high or low conditional probabilities of association with HHQ and NHQ are indicated with colors, and HHQ and NHQ itself are highlighted as larger balls. The plot is shown in two orientations for clarity. Download FIG S7, PDF file, 7.3 MB. Copyright © 2020 Raghuvanshi et al. 2020 Raghuvanshi et al. This content is distributed under the terms of the Creative Commons Attribution 4.0 International license.

### Multi-omics analysis of a CF mortality event.

**FIG 3:**
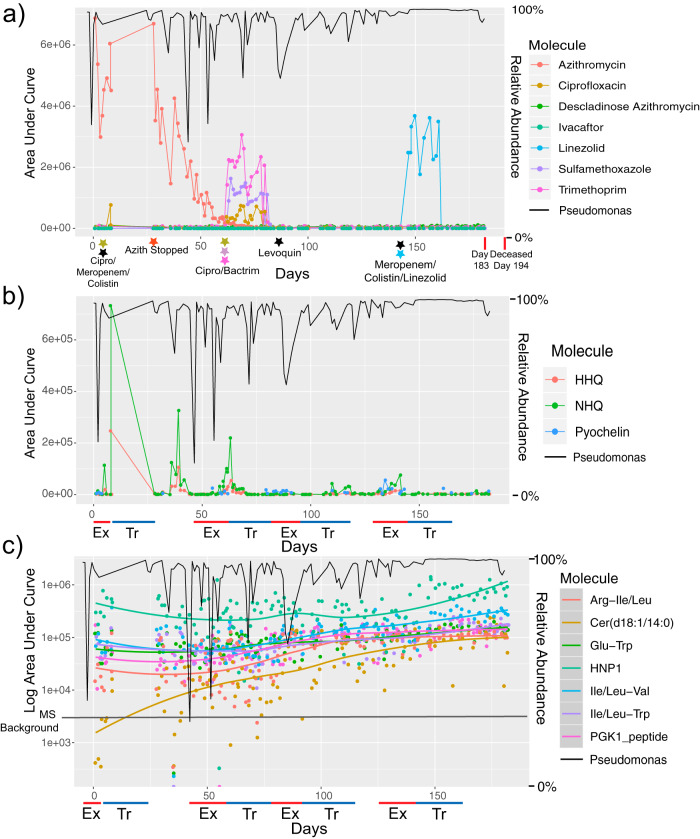
Microbial and metabolite changes prior to death of subject CF176. (a) Area under curve abundance of antibiotics detected in the metabolomics data from this subject (left *y* axis). The right *y* axis shows the abundance of *Pseudomonas* in the microbiome data plotted as a black line for reference. The *x*-axis data represent continuous time in days of sample collection for this subject. Black stars indicate antibiotics administered to the subject but not detected in the metabolomic data. (b) Area under curve abundance of Pseudomonas aeruginosa virulence-associated metabolites detected in the metabolomics data (left *y* axis). The right *y* axis shows the abundance of *Pseudomonas* in the microbiome data plotted as a black line for reference. (c) Log_10_ area under curve abundance of increasing levels of metabolites in CF176 through the collection time. The abundances of the metabolites are plotted along with the locally estimated scatterplot smoothing (LOESS) regression line for each molecule. The *Pseudomonas* relative abundance data are again plotted as a black line on the right *y* axis for reference. Ex, exacerbation; HNP1, human neutrophil peptide 1; PGK1, phosphoglycerate kinase 1; Tr, treatment.

The death of subject CF176 due to respiratory failure presented an opportunity to study the changes that occurred in the microbiome and metabolome as the fatal CFPE developed and subsequent treatment failed. Samples from CF176 were available for 118 (60%) of the 194 days prior to death during which the subject experienced four separate CFPEs and subsequent treatments with intravenous (i.v.) antibiotics, including the final course prior to death (Fig. 3a). These four exacerbations were experienced in a short time frame, indicating they may have been related, but each event was treated with a new course of different antibiotic combinations.

The microbial community was dominated by *Pseudomonas*, with a moderate increase in relative abundance through time (Pearson’s *r* = 0.323, *P* = 0.0004). There were spikes in the relative abundances of anaerobes and other oral bacteria observed, but these did not reliably correspond to CFPEs or their treatment in the first 60 days of collection (Fig. S8a and b). However, the onset of the third CFPE corresponded to an increase in the relative abundance of Haemophilus parainfluenzae which decreased upon treatment with levofloxacin (Levoquin) (Fig. S8a and b). The subject then entered a brief period of stability before experiencing a final fourth CFPE which featured another spike in the relative abundance of oral microbes (Fig. S8a and b). Treatment of this final CFPE consisted of intravenous (i.v.) meropenem, colistin, and linezolid followed by oral antibiotics in the 3 weeks before death. During this time, the *Pseudomonas* data reached a level of over 99.9% of sequenced reads and did not drop below 99.7% throughout the treatment course (Fig. 3a). No other known CF pathogens were detected in the final days of life (Fig. S8b). There were no increases in the relative abundances of anaerobes during the final treatments until 4 days before hospitalization at day 183. Unweighted UniFrac analysis suggested that the microbiome was changing through time (Fig. S8c and d), but weighted UniFrac analysis showed that the high relative abundance of *Pseudomonas* may have obscured the presence of a new infection in the final days of life (Fig. S8c and d).

10.1128/mSystems.00292-20.8 FIG S8 Microbiome changes in CF176, including in samples prior to death. (a) Microbiome profile of CF176 from both sputum sample collections merged. (CFPE and treatment states are shown, with stable periods left unhighlighted.) (b) Microbiome profile of CF176 with all *Pseudomonas* reads removed to show the underlying taxon profile. Samples with no taxa plotted amounted to 100% *Pseudomonas*. (c) Relative abundances of *Pseudomonas* with time in days in CF176 plotted with a locally estimated scatterplot smoothing (LOESS) smoothed line (Pearson’s correlations and *P* values are shown on the plot). (d and e) Unweighted UniFrac (d) and weighted UniFrac (e) data representing the CF176 sputum microbiome through time. The beta-diversity calculations were done with the cross-sectional (17) and longitudinal sample data included, but only samples from CF176 are shown in the main plots. The entire sample population PCoA plot is shown as an inset for reference, with subject CF176 samples highlighted as larger balls in the figure. The samples are colored by time in days. The first two axes represent the first and second principal components, and the third axis represents time in days. Download FIG S8, PDF file, 5.4 MB. Copyright © 2020 Raghuvanshi et al. 2020 Raghuvanshi et al. This content is distributed under the terms of the Creative Commons Attribution 4.0 International license.

The metabolomic data did not reveal strong changes in the months before death, though the effects of treatment were evident. For example, the level of i.v. antibiotics detected in the sputum corresponded to time of treatment, suggesting that the drugs were successfully diffusing to the target community. Metabolites from P. aeruginosa were also detected in this subject through time, but only sporadically, and did not coincide with CFPE, changes in *Pseudomonas* relative abundance, antibiotics, or death. HHQ, NHQ, and pyochelin were detected primarily early on in the sample collection and not in the final days of life (Fig. 3b). Molecules whose levels increased with time toward death were primarily peptides, human neutrophil protein 1 (HNP1), and a ceramide (Fig. 3c). The levels of these molecules increased gradually throughout the collection, with HNP1 increasing in the final 50 days of life.

Overall, the multi-omics analysis in this subject did not reveal any new CF pathogen infections, increased production of metabolites from *Pseudomonas*, or large changes in the overall metabolome/microbiome that could explain the subject’s death (Fig. 3; see also Fig. S8). Instead, the data reported here support a scenario where this subject had had a gradual increase in the relative abundance of *Pseudomonas* in sputum corresponding to increasing levels of metabolites associated with pulmonary inflammation leading up to death.

## DISCUSSION

This longitudinal study of six CF subjects provides further support for the notion of the presence of a dynamic microbiome and metabolome in CF sputum, as described by Caverly et al. (23), and provides new evidence for changes through the development and subsequent treatment of CFPEs. Collectively comparing across disease states, the level of microbial alpha-diversity and the log ratio of anaerobes to pathogens were decreased during antibiotic treatment for CFPEs but not between the stable and CFPE states. Comparing both of these measures with time showed that there was a linear increase approaching a CFPE treatment event, indicating that the microbiome changed as a CFPE developed. There was then a decrease in alpha-diversity upon initiation of treatment, and this remained lower without a temporal change through the treatment period. Linear changes with time around CFPE were not identified in the metabolome, likely due to the strong personalization in this data set. The signal for a reduction in microbial diversity and anaerobe abundance during antibiotic treatment supports previously described changes in a large cross-sectional study (5) and *in vitro* experiments (4). Thus, there is mounting evidence for changes between classic pathogens and anaerobic bacteria around CFPE events (4, 5, 13). However, our data also showed microbiome and metabolome dynamics during times of stability which was, at least in part, driven by maintenance antibiotics. Thus, future work is needed to determine if there are clinically relevant consequences of a changing microbiome during stability and if reduction of anaerobes during CFPE treatment corresponds to clinical response. This work will require extensive *in vitro* and *in vivo* experiments supported by mathematical models and clinical insights (23).

There are several important caveats for this study. First, at least a portion of the anaerobes detected in our samples might have been contaminants from the oral cavity during sputum expectoration (25). It is therefore possible that antibiotics were impacting the oral microbiota, which would then be reflected in contaminated sputum (26). The microbiome of subject CF189, however, where a single *Prevotella* ASV came to dominate the sputum microbiome for months, demonstrated that the anaerobe dynamics were unlikely to have stemmed from saliva contamination during expectoration, which would be expected to produce a more diverse and consistent assemblage of oral bacteria through time. Second, the grouping of samples into stable, CFPE, and treatment periods is not without its limitations. Subjects often receive oral antibiotics prior to CFPEs, and many cycle antibiotics as maintenance therapies, including the subjects in this study. Caverly et al. (23) identified maintenance antibiotics as a contributing factor to microbiome changes during stability. This study found a negative relationship between the abundance of the maintenance antibiotic trimethoprim in the metabolomic data and microbiome Shannon diversity during times of stability. This supports the notion that maintenance antibiotics may have contributed to dynamics during stability, although this relationship was not found with azithromycin and we cannot detect all maintenance drugs with our mass spectrometry methods. Third, sputum may be produced from different parts of the lung, which are known to have differing populations of microbiota (20). Nevertheless, even though sputum samples are not completely representative of the lung microbiome and may contain differing degrees of contamination from the oral cavity, the ease and low invasiveness of sputum collection enabled the large sample size in this study, which would have been impossible with more-invasive sample techniques, such as bronchoalveolar lavage, that more directly target lung microbiota. Another important point is that the subjects in this study were in different stages of disease progression, a factor which has been shown to impact the microbiome dynamics during CFPE therapy (5). There is evidence for this in our study as well, as late-stage subject CF176 had the most stable microbiome through time.

The collection of paired microbiome and metabolome data in our study enabled further investigation into the association between metabolites produced by Pseudomonas aeruginosa and ASVs of interest in the microbiome profiles. As reported previously from a cross-sectional study (17), in many samples that had large amounts of P. aeruginosa in the microbiome, there were small amounts or no metabolites from the bacterium detected. This may reflect dynamic changes in the growth rate and metabolite production of the bacterium in a subject through time, but this is only speculative, as many biological phenomena could explain this disparity, including disproportionate production of these compounds from different P. aeruginosa strains. Because microbiome/metabolite correlations are complicated by compositionality (30, 31), we applied a recently published method of computing metabolite-microbe conditional probabilities and did find an association between the *Pseudomonas* ASV and its quinolones. This method also identified negative associations between the P. aeruginosa quinolones and certain anaerobes, supporting the notion of the mutually exclusive dynamic between anaerobes and this bacterium that has been described previously (10). This negative association may represent antagonism between anaerobes and P. aeruginosa or contrasting niche occupancies, but future experiments are needed to identify any causal relationships behind these associations. An important limitation of our metabolomics methods is that the liquid chromatography-tandem mass spectrometry (LC-MS/MS) and extraction protocols used sample only a portion of the metabolome; additional extraction and mass spectrometry approaches will be needed to more comprehensively assess the sputum metabolomic makeup. It is also of note that other organisms can also produce some of these secondary metabolites; for example, *Burkholderia* spp. can produce rhamnolipids, pyochelin, and quinolones (32–34).

The unfortunate death of subject CF176 during this study provided an opportunity to study the changes in the microbial community and metabolome of sputum that occurred in the final stages of this disease. While a *Pseudomonas* ASV was the most highly abundant organism for most of the final 182 days of life, there were periods punctuated by increases in other microbes, particularly *Haemophilus*. There was no evidence of a new pathogen infection, a particularly marked microbiome change, or a dramatic increase in inflammation that might have explained this subject’s mortality. Instead, the data showed a progressive increase in the relative abundance of *Pseudomonas*, possibly driven by antibiotics administered for CFPEs, and a progressive increase in the relative abundances of metabolites and peptides associated with pulmonary inflammation. This type of mortality event may be common in late-stage CF, because many individuals exhibit a pathogen-dominated microbiome at this stage of disease progression (2, 17), but other multi-omics data surrounding mortality events besides this *n* = 1 case study are scarce. One report that is available on death associated with a severe CFPE implicated a new infection from Escherichia coli as a possible cause, though the conclusions were only speculative (35). As demonstrated here, multi-omic analyses enable insights into the complex interactions between host, microbe, and drugs through these tragic events of chronic disease. Perhaps most significantly, microbiome and metabolome data can now be generated in clinically relevant time frames (36), so this approach is a feasible route for future investigations in the clinic.

In conclusion, this study showed that the CF sputum microbiome is highly dynamic in some subjects through time, including during periods of clinical stability. During the development of a pulmonary exacerbation, there was an increase in microbial diversity corresponding to a relative increase of the ratio of anaerobes to pathogens, which then decreased during treatment. Thus, dynamics between classic pathogens and anaerobic bacteria around CFPE events may be important for therapeutic outcomes (4, 5, 10). Future studies that target CFPE therapy in a systematic manner are needed, particularly when the same antibiotic is repeatedly provided, to determine if any of the observed dynamics are predictable and if the reduction in anaerobe abundance during treatment corresponds to positive clinical outcomes. If so, this could lead to more precisely targeted and efficacious treatments for CF and improvements in subject quality and duration of life.

## MATERIALS AND METHODS

### Sample collection and clinical information.

The first sample collection comprised a total of 572 sputum samples from six CF subjects (see Data Set S1, sheet 2, in the supplemental material). These subjects were targeted due to recently poor health measures and lung function decline. A single Midea WHS-129C1 single-door chest freezer (3.5 cubic feet) was sent to the homes of each subject after consent to the study under institutional review board (IRB) research protocol no. 160078 (University of California [UC] San Diego). Subjects were asked to collect samples daily or as frequently as possible at their own discretion. One subject (CF146) collected twice daily for 15 of the 22 collection days, and data from these samples were averaged to represent a single sputum sample from that date. Samples were expectorated into 50-ml conical tubes that were then labeled by the subjects and stored in the freezers. At their convenience, subjects collected their samples and brought them on ice to the adult CF clinic at UC San Diego for permanent storage at –80°C. Samples were thawed once for aliquoting into cryovials and frozen again prior to multi-omics analysis.

After this initial sample collection, a secondary set of 22 samples was collected from subject 176 after this subject died. These samples were collected from the freezer retrospectively after death and shipped overnight on ice to the UC San Diego research laboratory for processing. Due to their priority, they were processed in the same manner as all others in the collection except that DNA and metabolites were extracted in triplicate wells of a 96-well plate and analyzed in triplicate. All subsequent plots and statistical analysis were done using means of data from the three triplicates for both the microbiome and metabolome. Samples from this secondary collection were integrated with the first set from the same subject as a case study of this single individual. However, these additional samples were not included in the statistical analyses describing the microbiome and metabolome dynamics from the initial collection, due to the potential for batch effects between runs that especially affect beta-diversity measures.

The samples were classified as exacerbation, treatment, or stable samples according to the clinical data obtained from the attending physician (Data Set S1, sheets 1 and 2). Exacerbations were defined as an increase in pulmonary symptoms associated with CF disease and the decision to administer intravenous or oral antibiotics to treat these symptoms for 21 days. The specific antibiotics administered for a CFPE were recorded as well as the start and end dates of each CFPE treatment course (see Data Set S1, sheets 1 and 2). The maintenance therapies given to each subject were also recorded, but the dates when these drugs were taken are not known (note that some maintenance therapies are detected in the metabolomics data, aiding interpretation of administration date; see the supplemental material). For analysis of antibiotic therapy effects on the microbiome and metabolome, samples were classified as “exacerbation” samples if they were collected within 14 days of an exacerbation diagnosis or as “treatment” samples if they were collected during the 21-day treatment course. If there was a change in the antibiotic chosen during the 21 days, this did not affect sample classification. Whether subjects were prescribed routine oral antibiotics prior to an exacerbation diagnosis was not considered in the classification, but to be included in the analysis as a CFPE, there had to be at least four samples collected prior to treatment.

### DNA extraction and 16S rRNA gene PCR.

A 200-μl aliquot of each sputum sample from a cryovial was added to a Thermo Scientific 96-well deep-well plate after thawing. The six plates containing these sputum samples were then subjected to DNA extraction performed with a Qiagen PowerSoil DNA extraction kit in 96-well format. The DNA extraction, PCR amplification, and barcoding of the V4 region of the bacterial 16S rRNA gene were completed according to the protocols for the earth microbiome project (37) described elsewhere (http://press.igsb.anl.gov/earthmicrobiome/protocols-and-standards/16s/). These protocols contain blank (no template) control samples that are used to identify background sequences in reagents or other contaminants.

### Microbiome data processing and analysis.

Raw sequence data were processed using Qiita (38) and were quality filtered following filtering recommendations (39) and processed by Deblur (40) to generate amplicon sequence variants (ASVs). Sequences were aligned in QIIME2 version 1.9.1 (41) using MAFFT in order to construct a phylogenetic tree using fasttree2. Taxonomy was assigned using q2-feature-classifier (42) against the 99% GreenGenes 16S rRNA reference database (version 13-8). Samples with fewer than 500 reads were removed, and the data were rarefied to 500 reads per sample, leaving 552 sputum samples for analysis. Information about the sequencing depth is available in the supplemental material (Data Set S1, sheet 7; see also Fig. S2 in the supplemental material). For analysis, QIIME2 version 2019.4.0 was used throughout. Core diversity metrics were computed using core metrics phylogenetic analysis for alpha- and beta-diversity indices. Based on their assigned taxonomy, ASVs were classified as either pathogens or anaerobes (Data Set S1, sheet 3). This classification was performed based on which highly abundant ASVs correspond to known classic CF pathogens targeted for antibiotic susceptibility in clinical laboratories and on other highly abundant ASVs in the entire data set that are known to represent obligate or aerotolerant anaerobes. The classified organisms collectively comprised 94.4% of total sequence reads in the data set. Organisms that did not fall into the classic pathogen or anaerobe categories (i.e., those that are not commonly considered CF pathogens but are not known anaerobes) were not included in calculations of pathogen/anaerobe ratios. The ASV assigned to the *Pseudomonadaceae* family was searched against the NCBI database with BLAST and verified to have 100% sequence similarity to Pseudomonas aeruginosa and other pseudomonads. It is therefore referred to as *Pseudomonas* throughout the manuscript. Similarly, the ASV assigned to *Escherichia* had 100% identity to *Escherichia* spp., but the species was not identified due to high levels of similarity in the 16S rRNA gene V4 region within this group.

### Metabolite extraction and metabolomics.

Metabolites were extracted using a modified version of the 96-well plate extraction procedure described previously by Quinn et al. (19). Briefly, a 200-μl aliquot of each sputum sample was added to a Thermo Scientific 96-well deep-well plate for metabolite extraction. First, 300 μl of ethyl acetate was added to the sputum, subjected to vortex mixing, and allowed to extract at room temperature overnight. The plate was then spun at 2,000 × *g* in a tabletop centrifuge, and then 200 μl of the ethyl acetate layer was removed and dried in the plate overnight. Next, 300 μl of methanol was added to the remaining sputum, subjected to vortex mixing, and then extracted overnight at 4°C. The plate was spun again to separate particulates from the methanol extract, and 200 μl was added to the dried ethyl acetate extract. The extracted metabolites were then diluted 1:2 in methanol spiked with 2 μM ampicillin as an internal standard. Control blank samples also went through the entire extraction process but without sputum to allow for removal of background signals from the solvents and mass spectrometer. This extract was analyzed by injection into a Bruker Daltonics Maxis Impact II LC-MS/MS system according to the mass spectrometry protocols described previously by Quinn et al. (17). Briefly, a 20-μl injection volume was separated using a Kinetex 1.7-μm-pore-size C_18_ ultraperformance liquid chromatography (UPLC) column (50 by 2.10 mm) with a linear gradient of 2:98 water to acetonitrile progressing to 98:2 acetonitrile to water for a 14-min run. The data were then converted to the .mzXML format for metabolite quantitation and annotation with GNPS. Volatile metabolites, those that are strongly nonpolar, and those that ionize only in negative mode are not likely to be detected with this method.

### GNPS analysis and metabolite feature finding.

Data from the mass spectrometer expressed in Bruker .d format were first converted to the .mzXML format and then uploaded to the GNPS database and data analysis server (gnps.ucsd.edu). The data are publicly available as MassIVE data set number MSV000082667. Molecular networks were built on GNPS with the following parameters: mass tolerances of 0.03 Da, cosine score of 0.65, minimum number of matched fragment ions of 4, and minimum cluster size of 3 spectra. Library searching parameters were the same with a cosine score of 0.65 and a minimum number of matched peaks of 4 (a list of library hits is available in Data Set S1, sheet 4; these are level 2 according to the metabolomics standards initiative [43]). Metabolites that were annotated without direct GNPS library hits were identified through propagating annotations through the GNPS networks and inspection of MS/MS spectral patterns (Data Set S1, sheet 7; level 2 according to a previously described classification system [43]). The molecular network used for analysis of the sputum data in this project is available at https://gnps.ucsd.edu/ProteoSAFe/status.jsp?task=e9e9002371794bedbf8faf38e632a3f4. The secondary data set had the same parameters used, but the molecular network is available at https://gnps.ucsd.edu/ProteoSAFe/status.jsp?task=70f4483662db4489821a3773783f9641.

Area under the curve abundances of metabolite features were quantified using mzMine2 software (44). The chromatograms were built with the following parameters: MS^1^ noise level of 5000 counts, MS^2^ noise level of 200 counts, minimum time span for chromatograms of 0.01, minimum height of 10,000, and a 10-ppm mass tolerance. Chromatograms were deconvoluted, and the isotope peaks were grouped to remove redundancy. The data were aligned with a retention time tolerance of 0.2 min and an *m*/*z* tolerance of 0.03 or 10 ppm. Metabolites detected in blanks and background controls were removed prior to statistical analysis.

### Statistical analysis.

The weighted UniFrac (45) distance matrix was computed in QIIME2 to project the sample similarities of the microbiome data in a three-dimensional principal-coordinate analysis (PCoA) plot using EMPeror (46). Similarly, the Bray-Curtis distance matrix was computed in R on the metabolomics feature table and visualized with a PCoA plot (the UniFrac distance cannot currently be calculated on metabolome data). PERMANOVA testing on the beta-diversity measures was done with subject source and disease state classifiers in R using the vegan package. Testing with respect to disease state accounted for subject source as a covariate using the “strata” function. The microbiome data were also plotted in PCoA space with the third axis as time in days, to visualize the changing microbiome through time (see Video S1 in the supplemental material). The UniFrac and Bray-Curtis distance values were quantified within each subject through time using a method similar to that described previously by Caverly et al. (23). All pairwise comparisons of each sample to all others from the same subject were plotted as notch plots, and the percentages of samples outside 1.5× the interquartile range (IQR) were calculated. In addition, the percentages of samples within a subject with a distance value above 0.6 were reported to assess those that were highly different from the rest. To provide relevant comparisons of these numbers, the same calculations were then done on a publicly available cross-sectional CF sputum data set analyzed with microbiome and metabolome methods published previously (17).

The same respective distance measures were used to test for significance of within-subject and between-subject beta-diversity by computing the mean distances of each sample from a subject to all samples from other subjects (between distances) and comparing the results to the distances of each sample from a subject to all others from the same subject (within distances). Comparisons within and between subjects for both microbiome and metabolome were tested for significance using a linear mixed-effects model (LME) from the *lmer4* package in R with subject as a random effect. The alpha- and beta-diversities of microbiome and metabolome samples were also compared across subjects using a Kruskall-Wallis test for significant differences. A *post hoc* Dunn test adjusted for multiple comparisons performed with the Benjamini-Hochberg method was used to compare pairwise significance data corresponding to microbiome and metabolome beta-diversity between individuals.

To determine whether the levels of microbiome and metabolome beta-diversity differed between disease states, all samples from each subject were compared to each other within each disease state in a pairwise manner using the weighted UniFrac distance (fixed effect). These comparisons were limited, however, to samples collected within 21 days of each other, to minimize the large variations that might have occurred through time and to normalize the time across disease state classes (i.e., not to compare exacerbations or stable samples collected months apart). These UniFrac distance values were then compared using an LME model with subject source as a random effect. The *post hoc* pairwise comparisons across disease states were then tested with a Tukey’s test on the mixed model using the Simultaneous Inference of General Parametric Models (multcomp) package in R. Alpha-diversity changes (Shannon index) and the log ratio of anaerobes to pathogens across different disease states were also tested with the same LME method. Changes in total bacterial load in the three disease states were tested with the same LME model.

For longitudinal comparisons of changes in the microbiome and metabolome with time through CFPE development and treatment, LME models were used with subjects set as random effects to account for the different sample numbers from the six subjects. All models were run with the *lmer4* package in R statistical software. Statistical significance was computed using Satterthwaite’s degrees of freedom method from the *lmerTest* package in R. The linear trend in Shannon diversity (fixed effect) and the log ratio of the relative abundance of anaerobes compared to pathogens (fixed effect) were modeled using this method. For further support, the trend in Shannon diversity through time during CFPE treatment was also modeled and tested with mixed-effects models with linear splines by the use of *lme* in the *nlme* R packages. The temporal trajectories were assessed with a linear spline (or broken stick) model and then tested using “Eigen” and S4 (*lme4*) to determine whether there was an effect of subject status on the Shannon diversity (fixed effects). Subjects and days relative to CFPE over time were also considered random effects in this model. The same linear mixed-model approach was used to test the correlation between the abundance of a maintenance antibiotic from the metabolomic data and Shannon diversity from the microbiome data in the same samples.

A random forests classification model was used to identify metabolites that separated the stable, CFPE, and treatment groups. This model was run using the randomForest package in R with 5,000 trees and 59 variables tried at each split and with stratification due to differential sample numbers in each disease class.

Microbe-metabolite cooccurrence probabilities were calculated using MMvec, a neural network approach trained to predict metabolite abundances given the presence of a single microbe (29). This model was trained using three principal axes with a batch size of 10,000 and 10,000 epochs. MMvec performs cross-validation by leaving out samples and evaluating how well the metabolites can be predicted solely from the microbe abundances in the hold-out samples. The cross-validation error values converged, suggesting little overfitting. We first preprocessed the data by removing features/ASVs that appeared in fewer than 10 samples. Five samples were selected for hold-out testing, where the model predicted the metabolite abundances from the microbe abundances in these samples.

### Data accessibility.

The microbiome data were deposited in the Qiita (38) database as project number 11400, and the second data set was deposited under accession no. 11433. The data are also publicly available at the European Bioinformatics Institute under accession no. ERP119164.

10.1128/mSystems.00292-20.10 VIDEO S1 PCoA plot of microbiome data from four subjects in this study (CF066, CF176, CF189, and CF318) coanalyzed with a cross-sectional cohort (Qiita study ID11284, published previously [17]). The cross-sectional data are plotted in the first two principal components, and the samples are colored according to the percentage of *Pseudomonas* from 0% (white) to 100% (red). Time in days is indicated on the third axis, and the movement through the PCoA space of the four subjects is animated in Video S1. As Video S1 plays, the viewer can watch each subject move through the CF microbiome PCoA space. There were times when the subjects remained in the high-*Pseudomonas* region of the plot and times when they shifted away from that region. Download Movie S1, MOV file, 10.1 MB. Copyright © 2020 Raghuvanshi et al. 2020 Raghuvanshi et al. This content is distributed under the terms of the Creative Commons Attribution 4.0 International license.
