# Supplementary material for: High-Resolution Longitudinal Dynamics of the Cystic Fibrosis Sputum Microbiome and Metabolome through Antibiotic Therapy
Source: mSystems. 2020 Jun 23;5(3):e00292-20. doi: 10.1128/mSystems.00292-20 (PMC7311317; doi:10.1128/mSystems.00292-20)
Supplement: FIG S1 [file mSystems.00292-20-sf001.pdf]

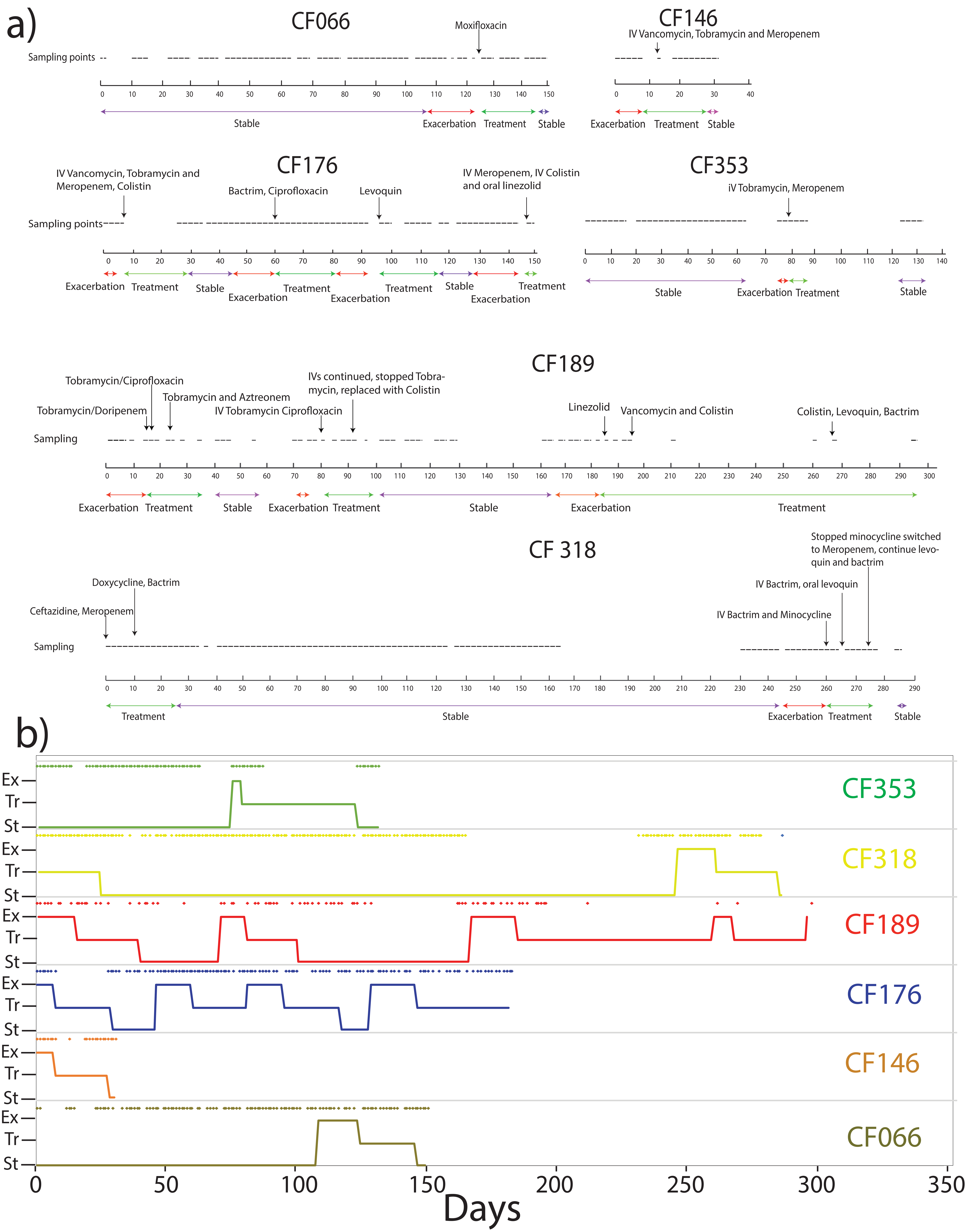
a)
CF066
Moxifloxacin
Sampling points
Stable
Exacerbation
Treatment
Stable
CF146
IV Vancomycin, Tobramycin and Meropenem
Exacerbation
Treatment
Stable
CF176
IV Vancomycin, Tobramycin and Meropenem, Colistin
Bactrim, Ciprofloxacin
Levoquin
IV Meropenem, IV Colistin and oral linezolid
Sampling points
Exacerbation
Treatment
Stable
Exacerbation
Treatment
Exacerbation
Treatment
Stable
Treatment
Exacerbation
CF353
iV Tobramycin, Meropenem
Stable
Exacerbation
Treatment
Stable
CF189
Tobramycin/Ciprofloxacin
Tobramycin and Aztreonem
Tobramycin/Doripenem
IVs continued, stopped Tobramycin, replaced with Colistin
IV Tobramycin Ciprofloxacin
Linezolid
Vancomycin and Colistin
Colistin, Levoquin, Bactrim
Sampling
Exacerbation
Treatment
Stable
Exacerbation
Treatment
Stable
Exacerbation
Treatment
CF 318
Stopped minocycline switched to Meropenem, continue levoquin and bactrim
IV Bactrim, oral levoquin
IV Bactrim and Minocycline
Doxycycline, Bactrim
Ceftazidine, Meropenem
Sampling
Treatment
Stable
Exacerbation
Treatment
Stable
b)
Ex
Tr
St
CF353
CF318
CF189
CF176
CF146
CF066
0
50
100
150
200
250
300
350
Days
