# Supplementary figures and images for: High-Resolution Longitudinal Dynamics of the Cystic Fibrosis Sputum Microbiome and Metabolome through Antibiotic Therapy
Source: mSystems. 2020 Jun 23;5(3):e00292-20. doi: 10.1128/mSystems.00292-20 (PMC7311317; doi:10.1128/mSystems.00292-20)

a)

% of Total Ion Current

All Mass Spectral Features

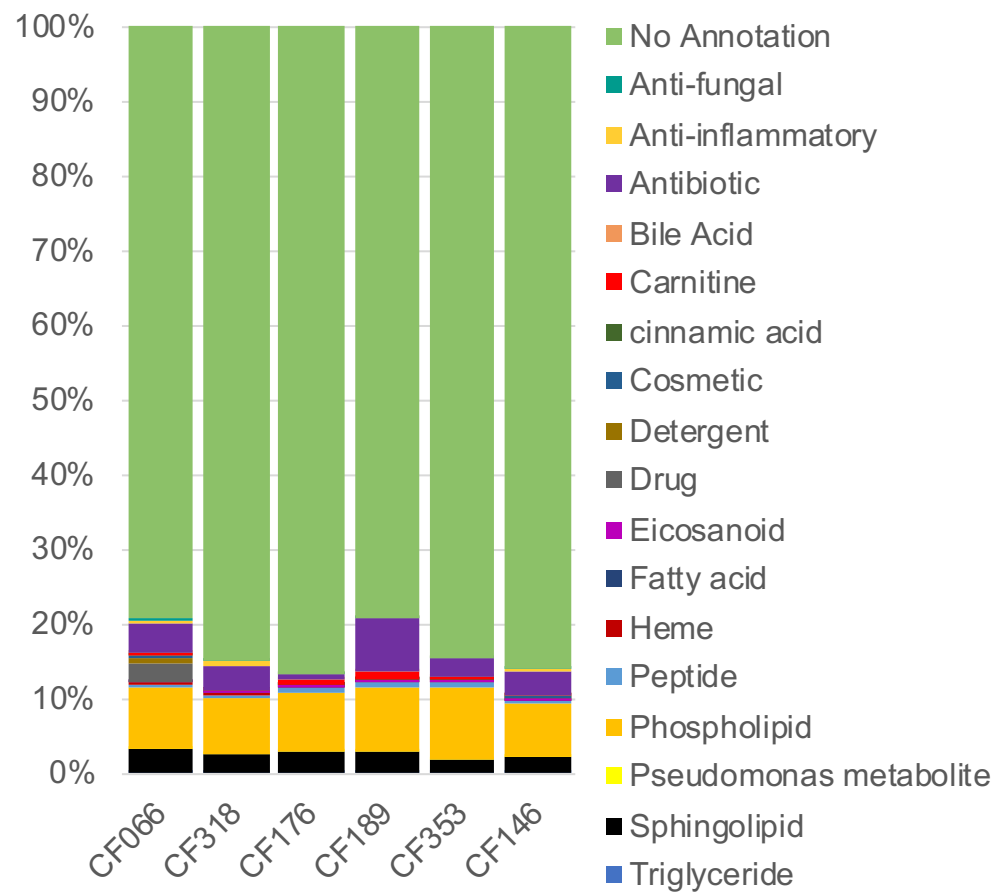

Annotated Mass Spectral Features

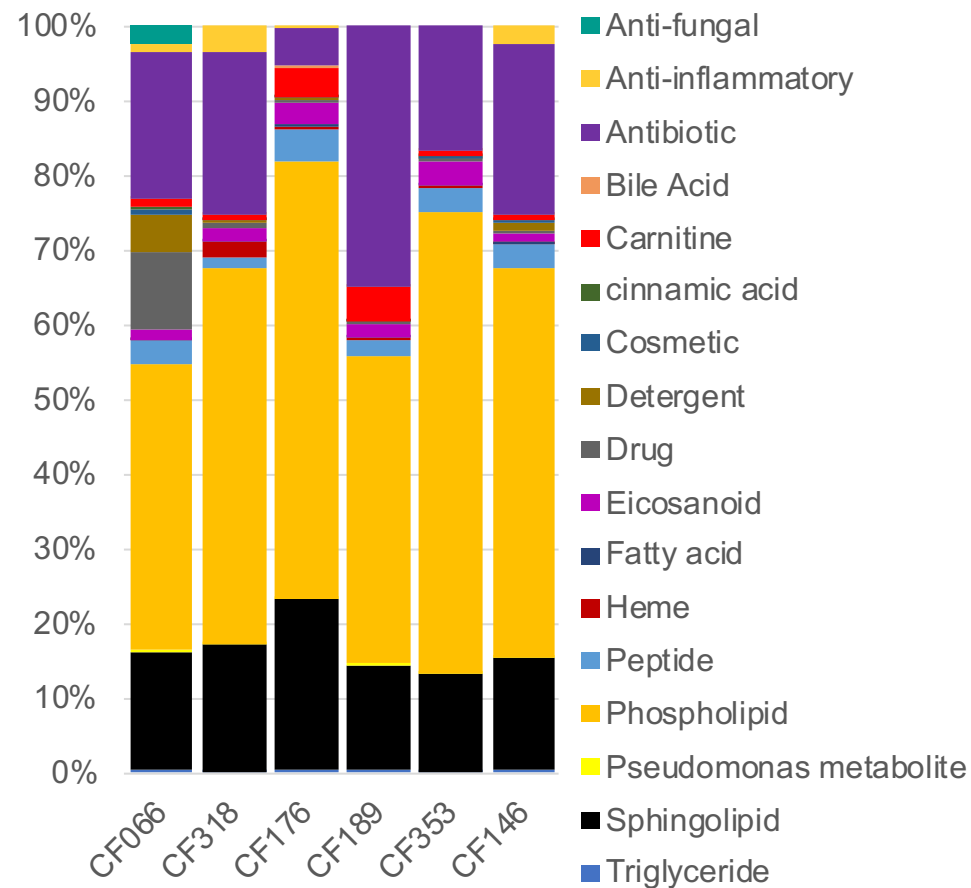

b)

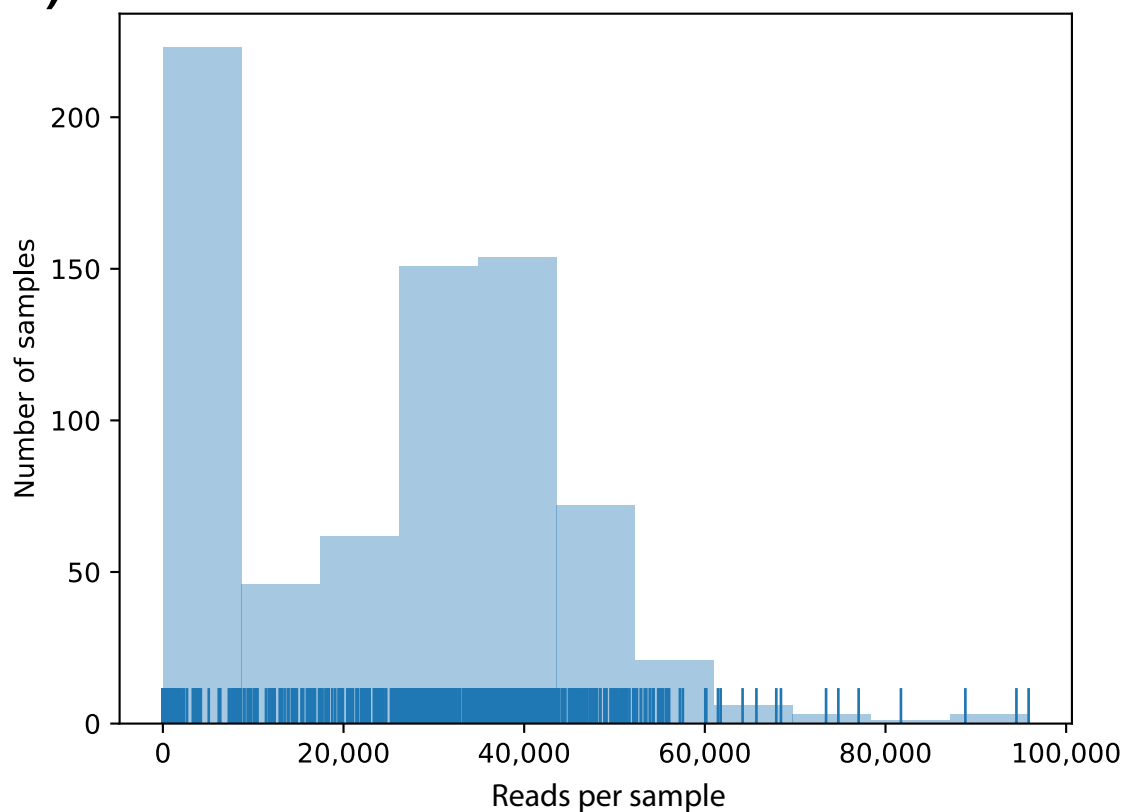

Supplement: FIG S2 [file mSystems.00292-20-sf002.pdf]

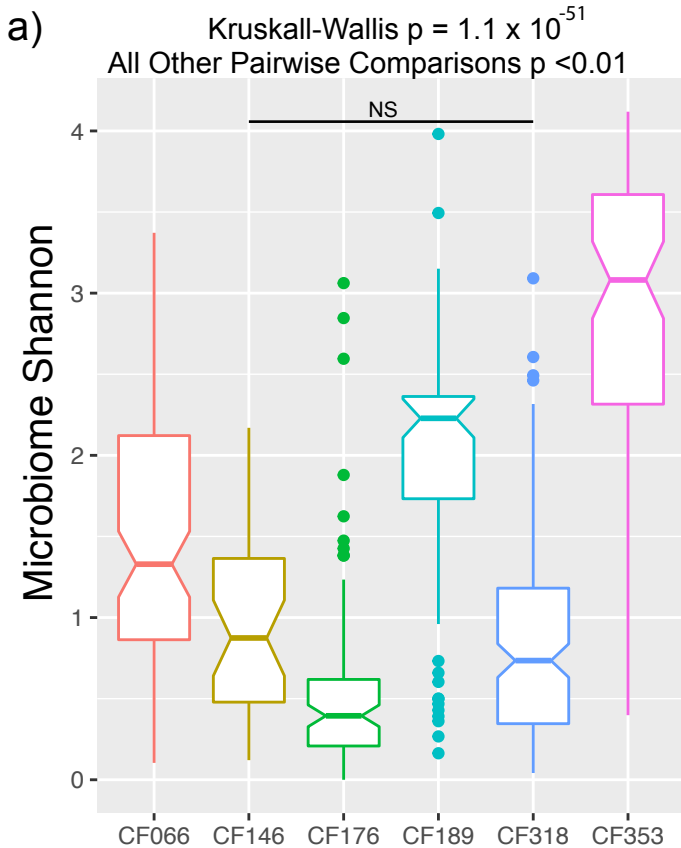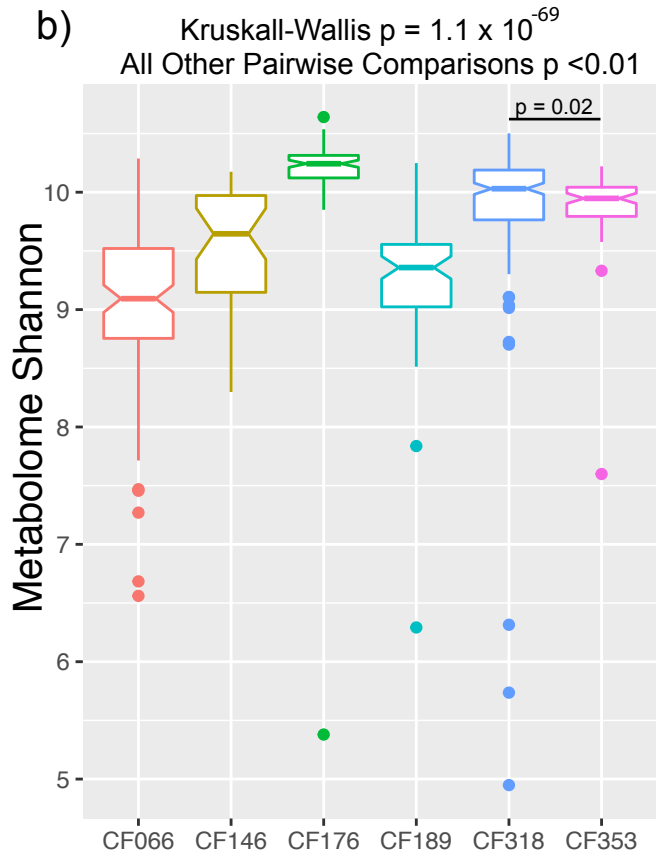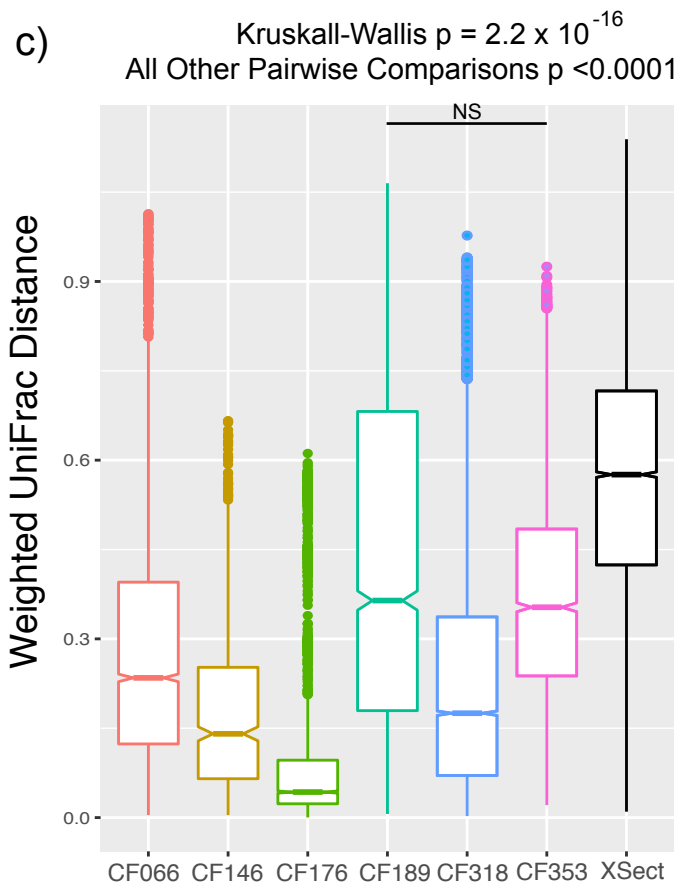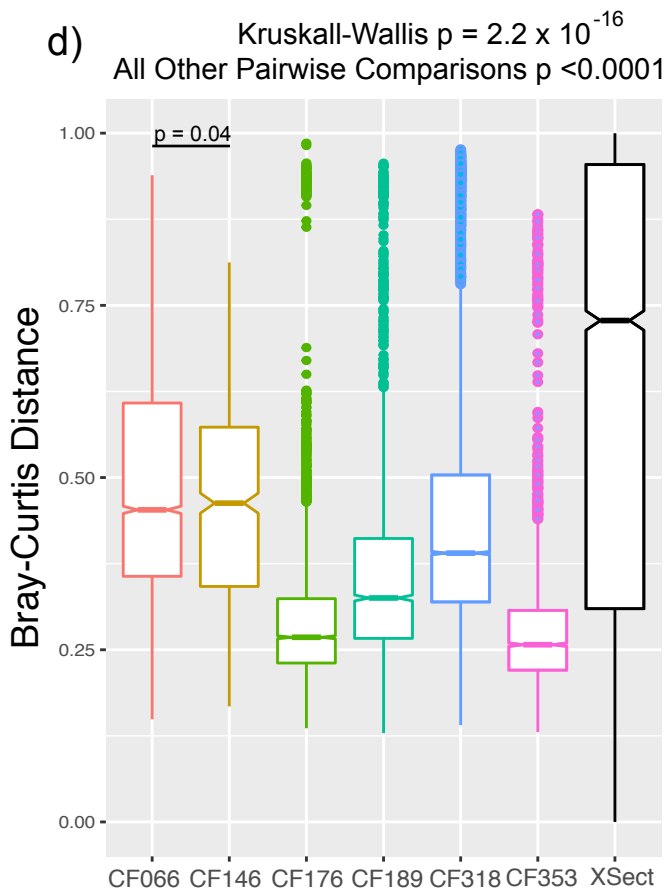

Supplement: FIG S3 [file mSystems.00292-20-sf003.pdf]

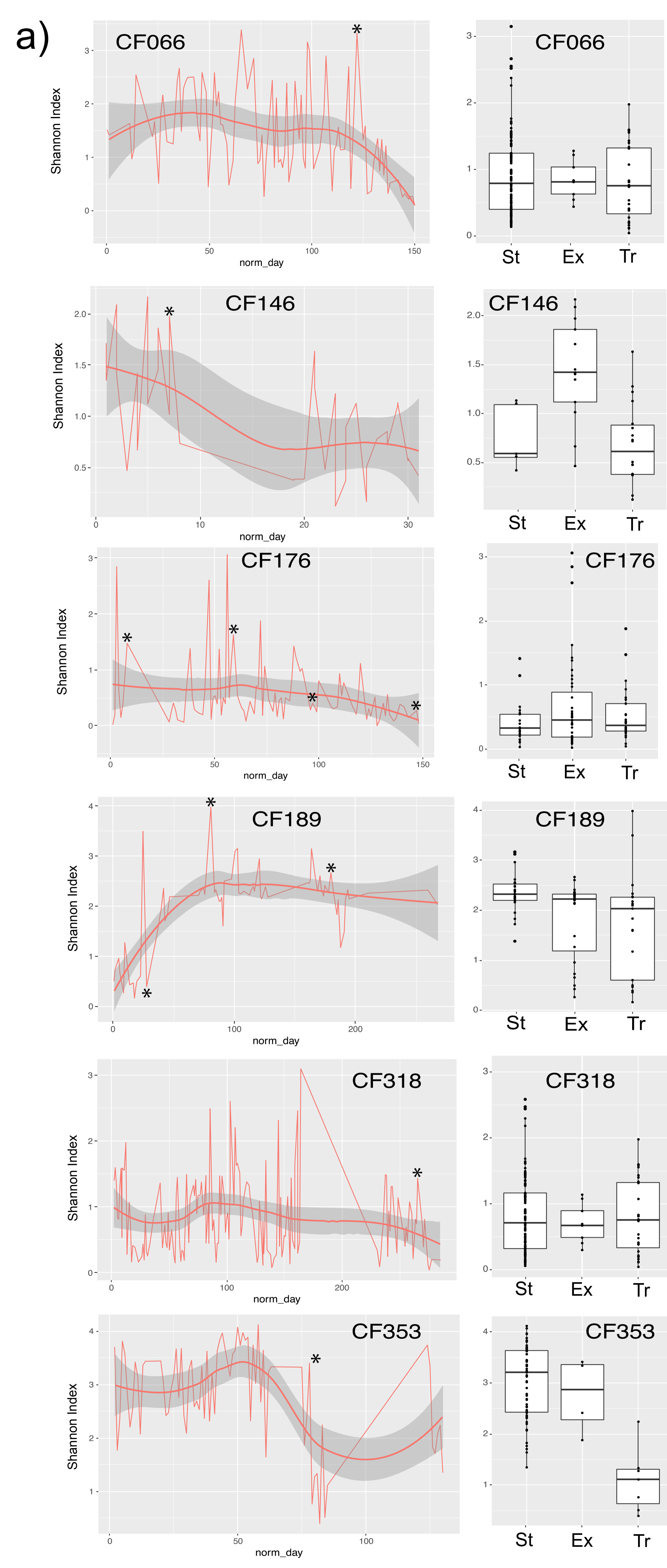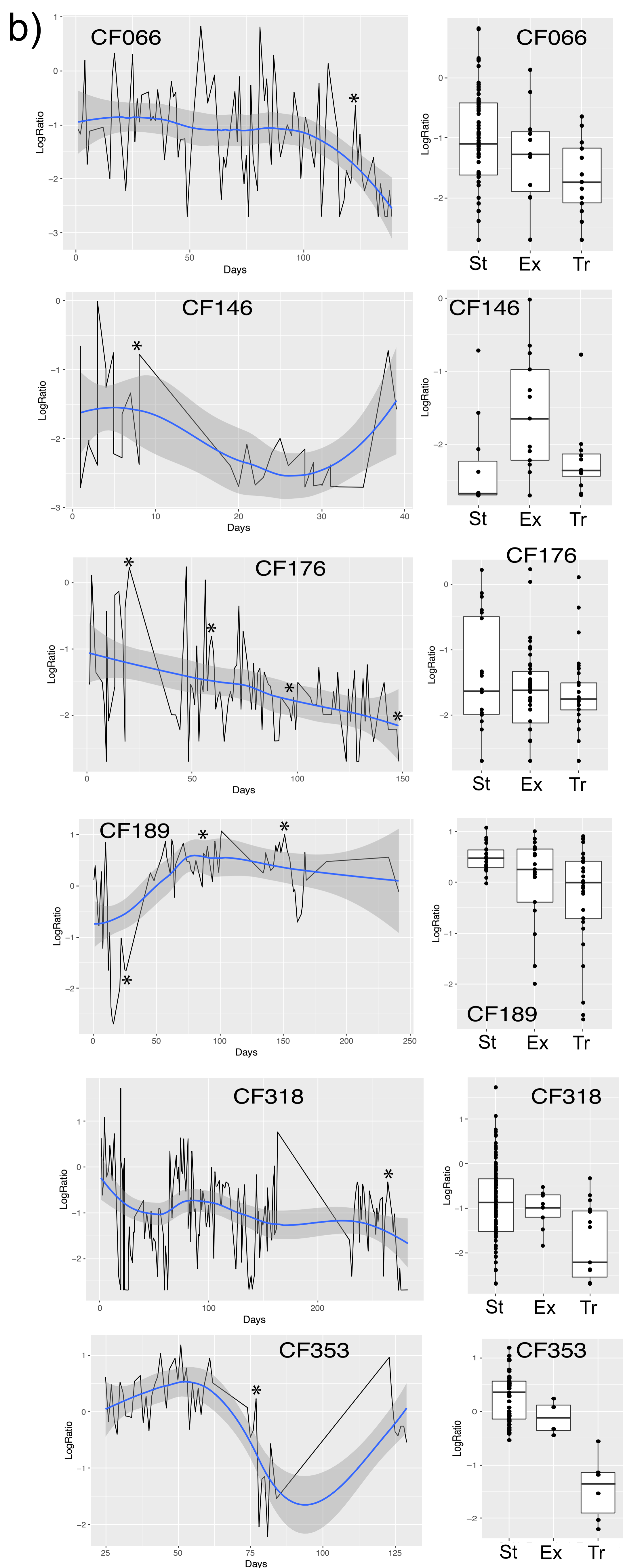

Supplement: FIG S4 [file mSystems.00292-20-sf004.pdf]

# Microbiome

Distances to CF176

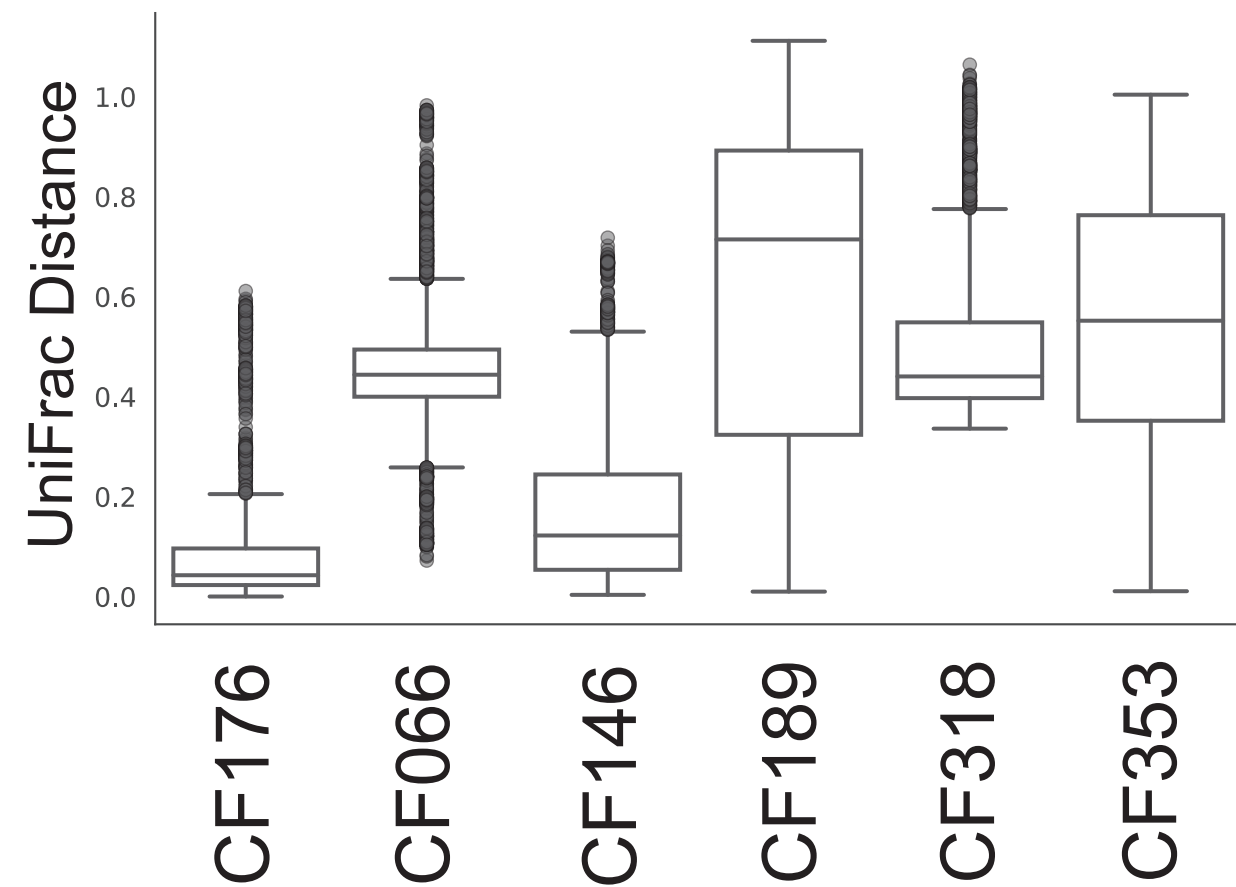

# Metabolome

Distances to CF176

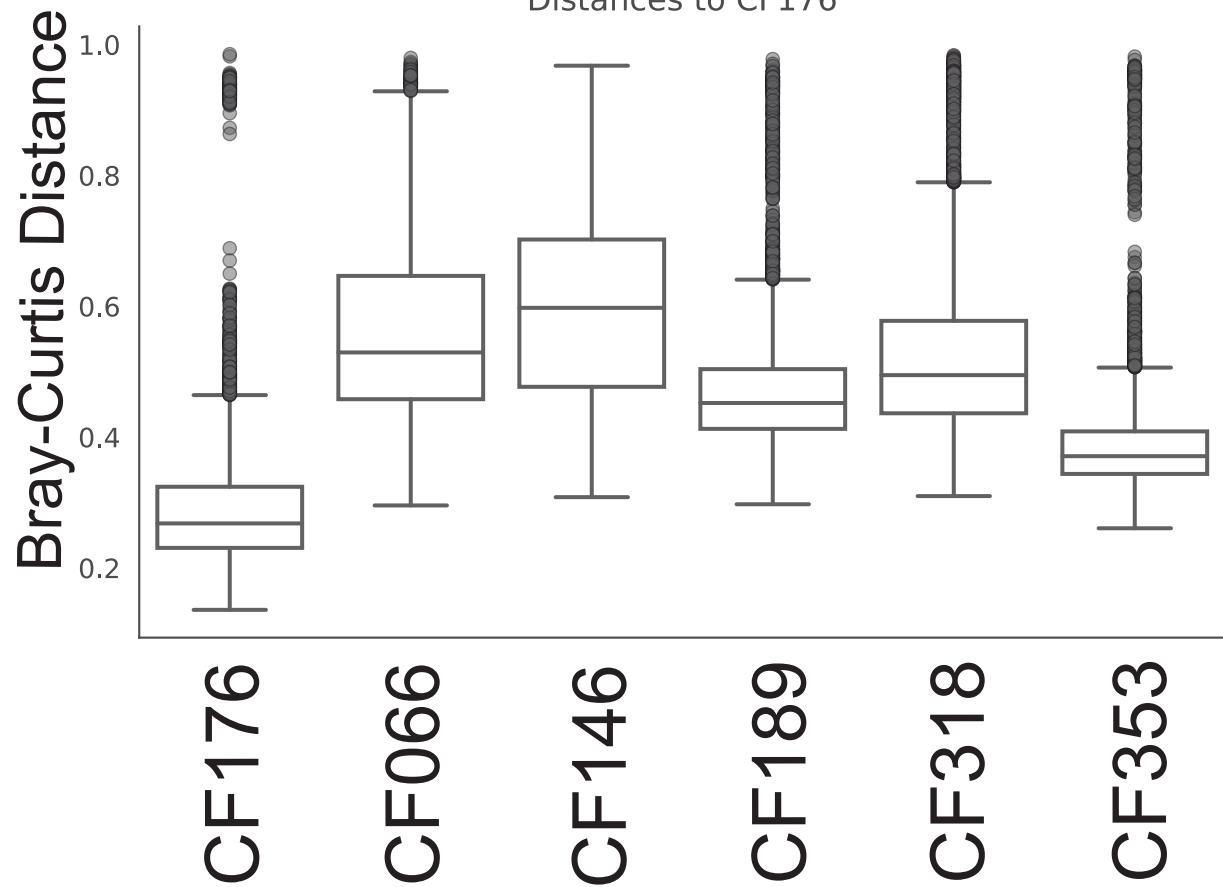

Supplement: FIG S5 [file mSystems.00292-20-sf005.pdf]

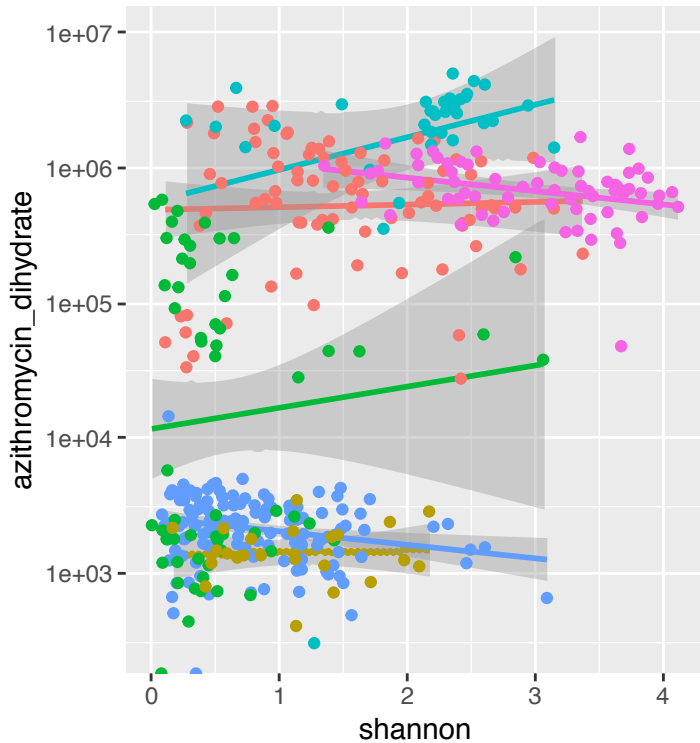

$\rho = 0.35$   
LMM  $p = 0.489$

deidentified\_patient\_number

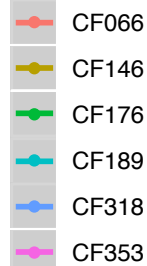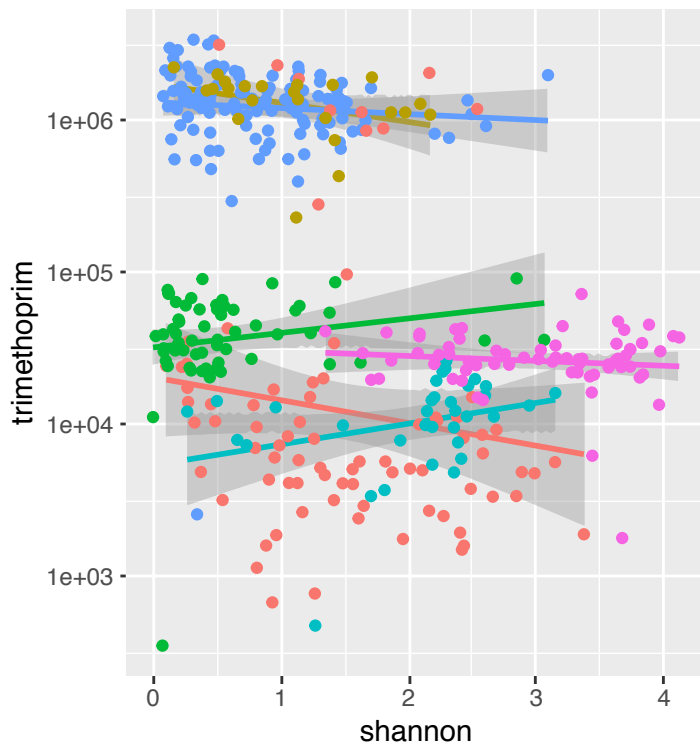

$\rho = -0.39$   
LMM  $p = 0.0048$

deidentified\_patient\_number

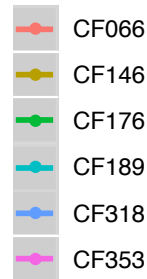

Supplement: FIG S6 [file mSystems.00292-20-sf006.pdf]

a)

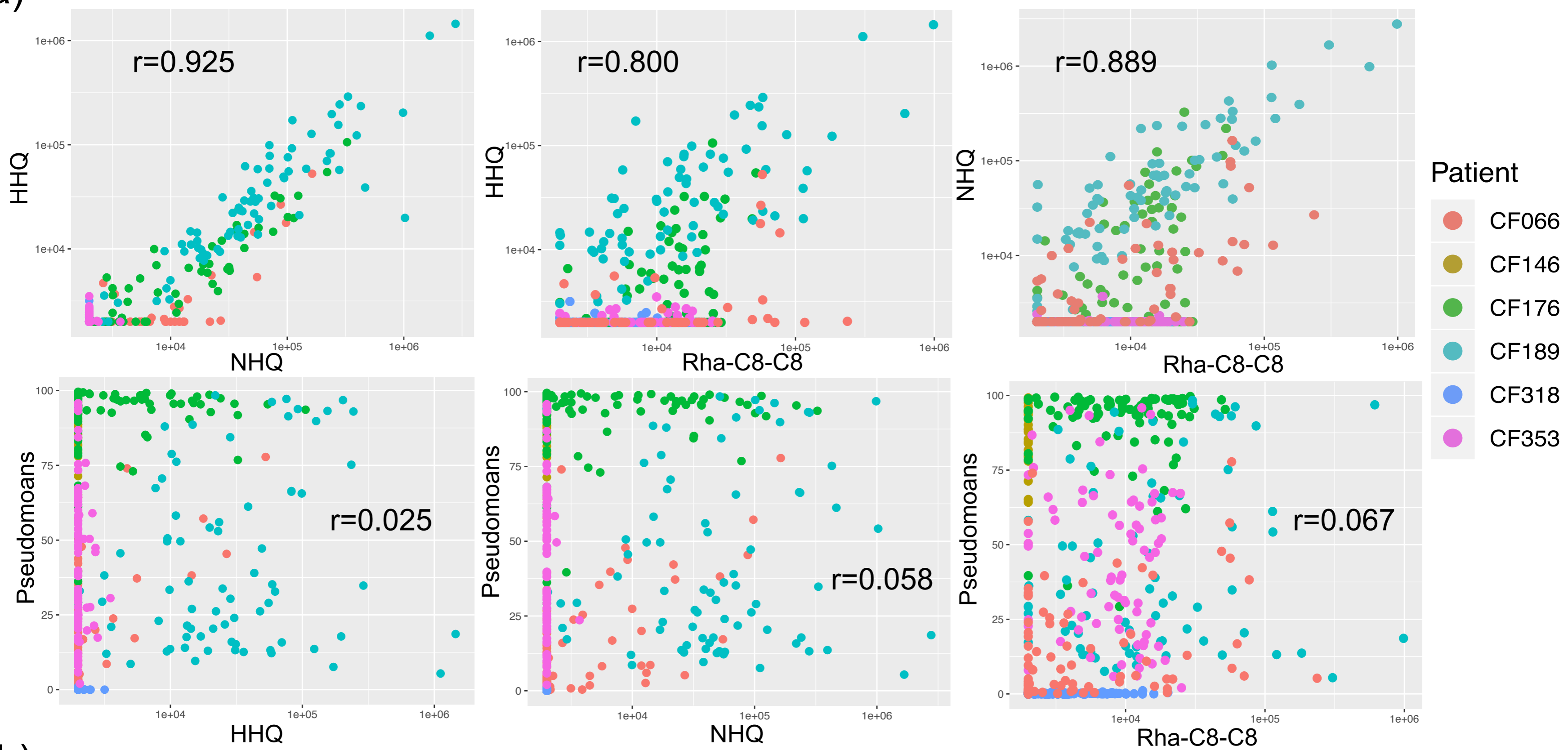

b)

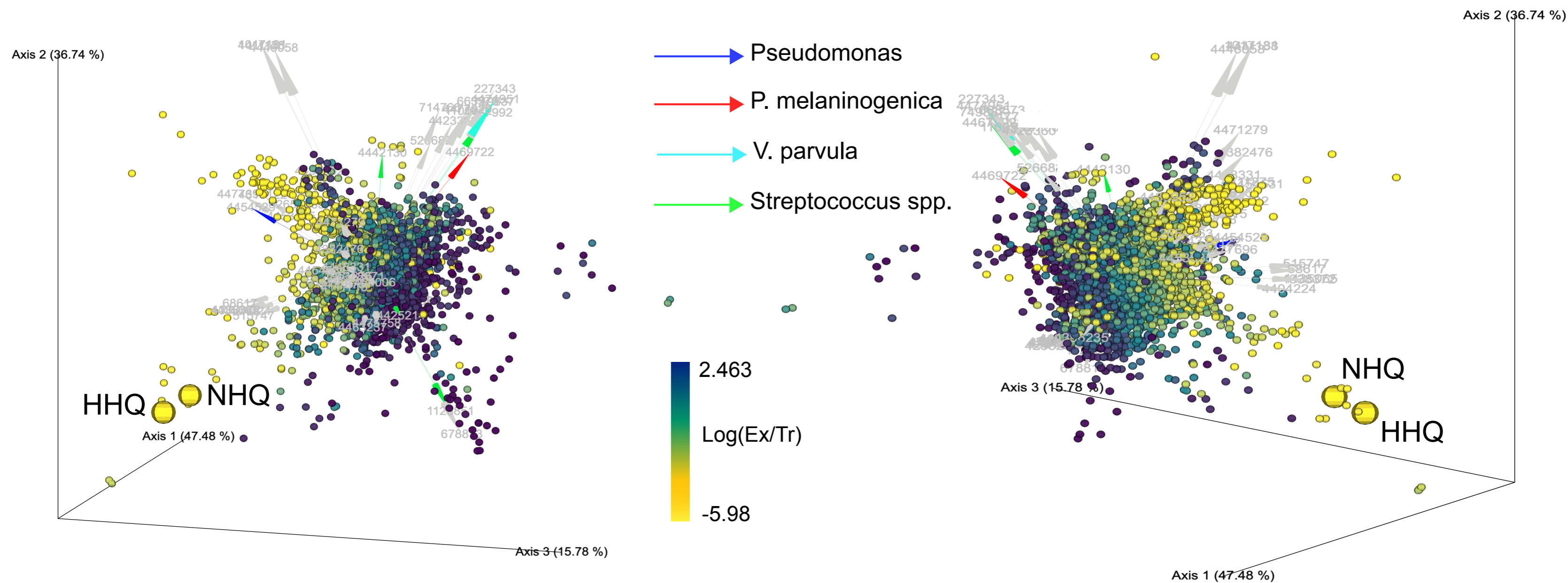

Supplement: FIG S7 [file mSystems.00292-20-sf007.pdf]

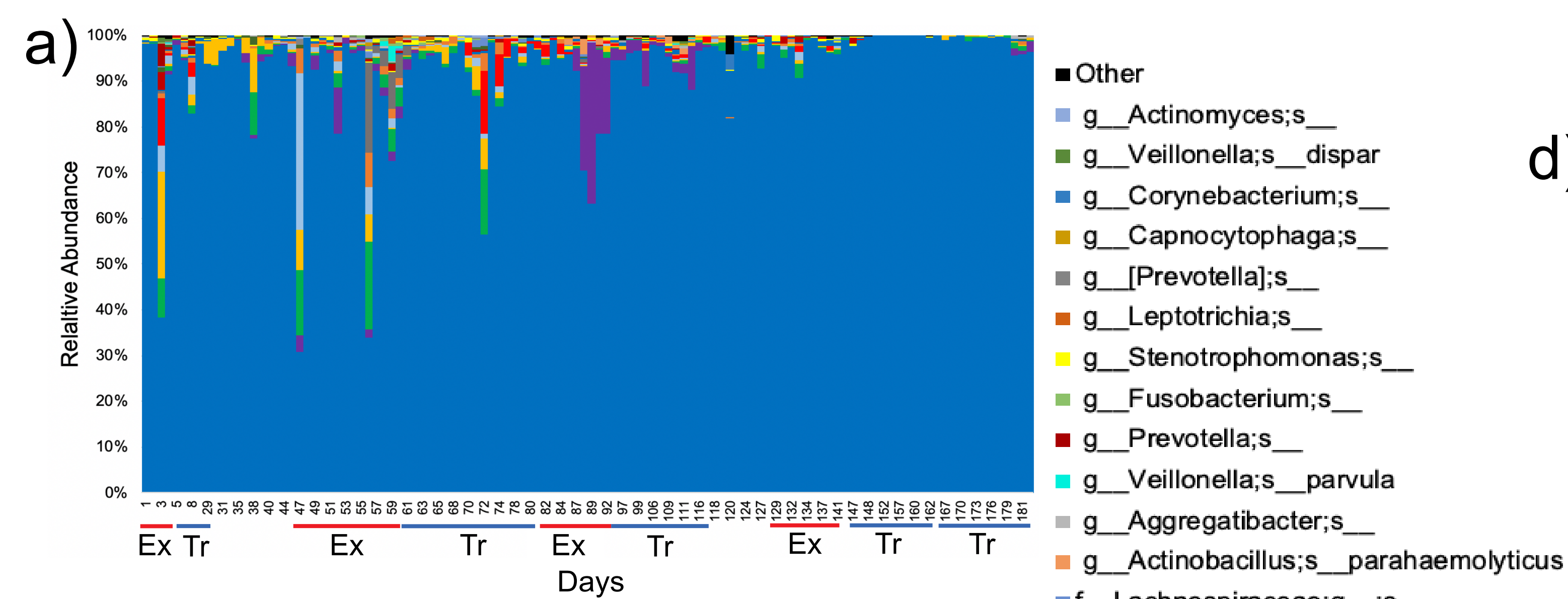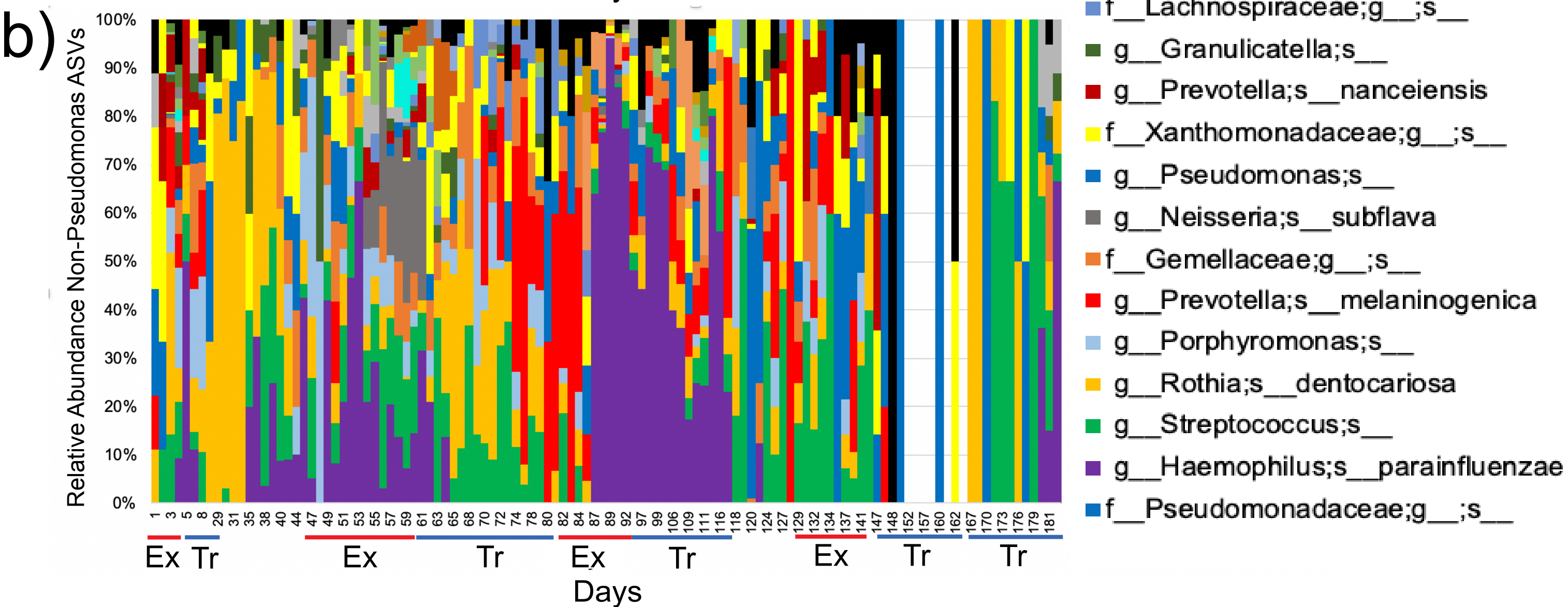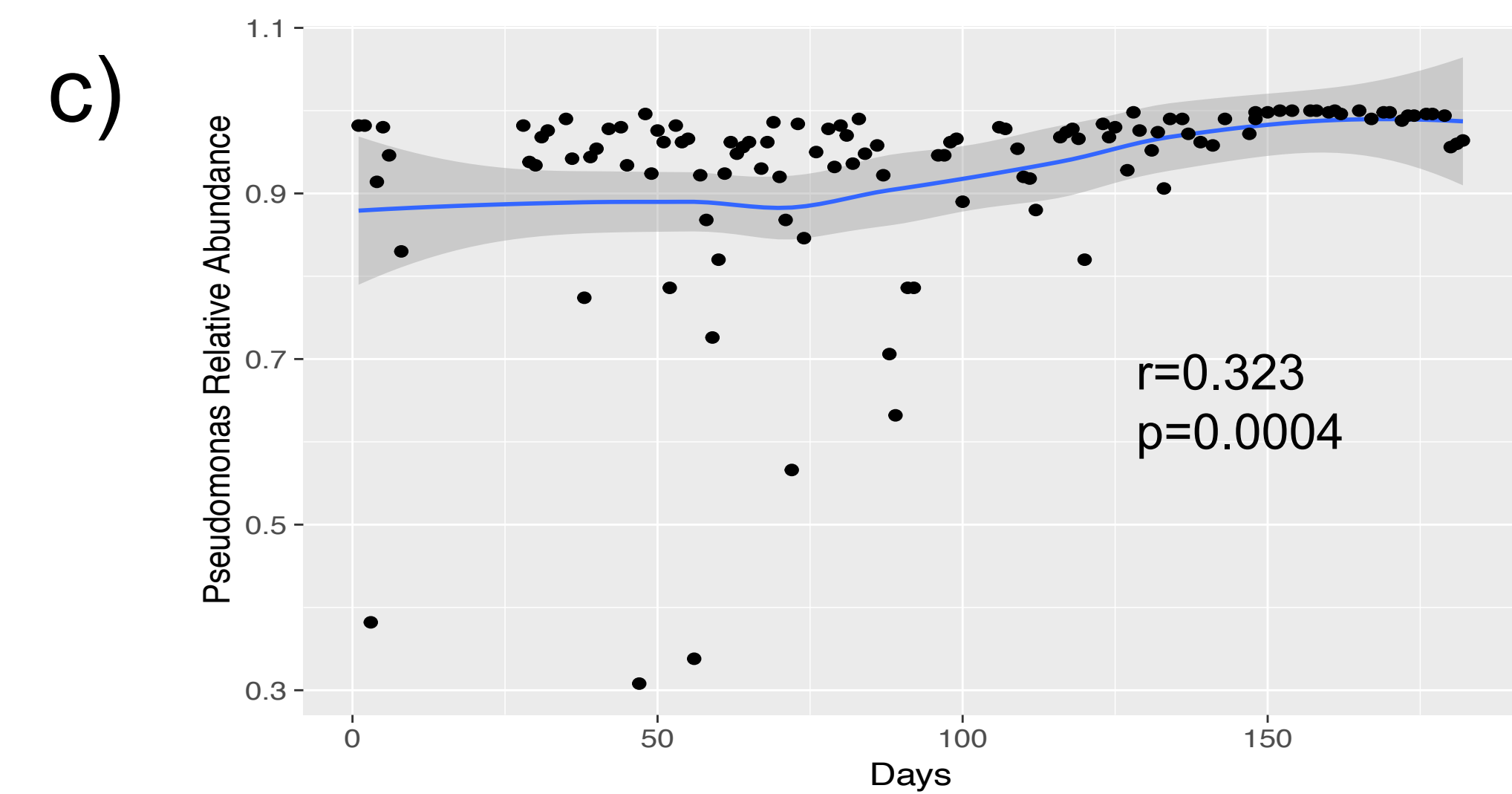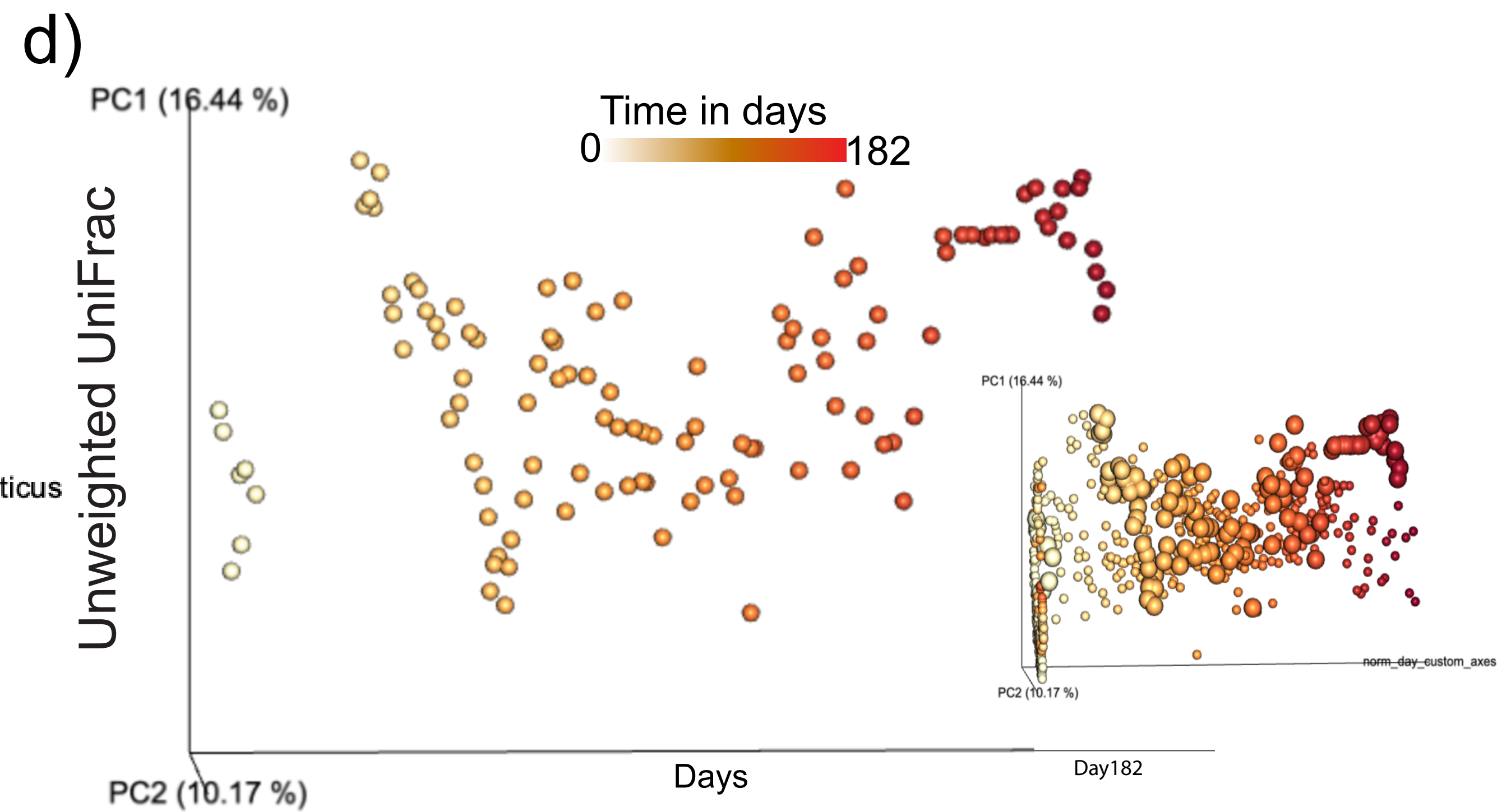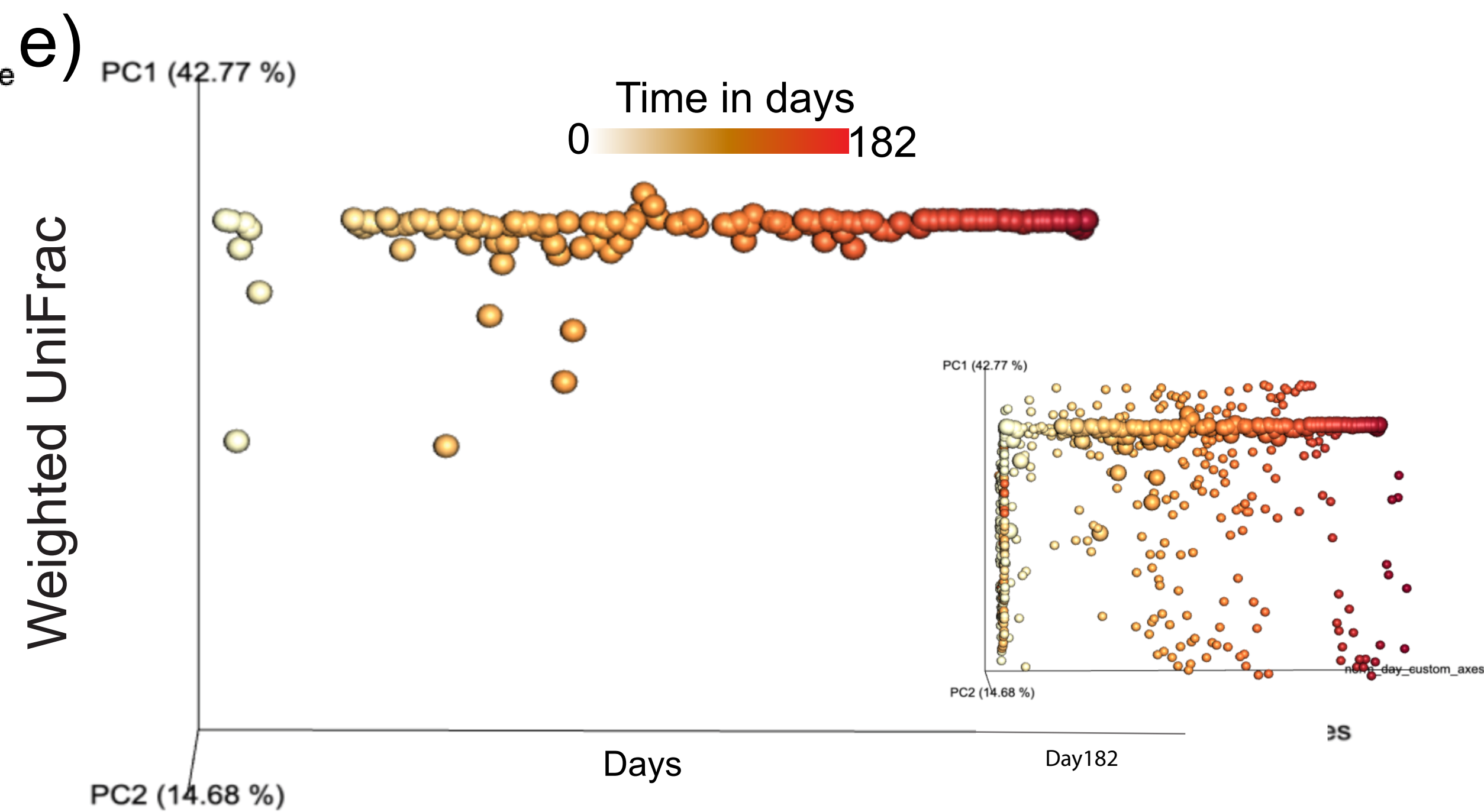

Supplement: FIG S8 [file mSystems.00292-20-sf008.pdf]
